# Engineering Access to Stereoirregular Polymer Microstructures Enables Improved Processability of Microbial Poly(3-hydroxybutyrate)

**DOI:** 10.1021/acs.biomac.6c00971

**Published:** 2026-06-12

**Authors:** Marcel Mayer, Julian Helberg, Kai Stirnweiß, Navaneeth Shiva Kumar, Daniel Van Opdenbosch, Doris Schieder, Cordt Zollfrank, Volker Sieber

**Affiliations:** † Chair of Chemistry of Biogenic Resources, Campus Straubing for Biotechnology and Sustainability, 9184 Technical University of Munich, Straubing 94315, Germany; ‡ Chair of Biogenic Polymers, Campus Straubing for Biotechnology and Sustainability, Technical University of Munich, Straubing 94315, Germany; § SynBioFoundry@TUM, Technical University of Munich, Straubing 94315, Germany; ∥ Center for Microplastic Prevention, Technical University of Munich, Straubing 94315, Germany; ⊥ Catalysis Research Center, Technical University of Munich, Garching 85748, Germany

## Abstract

Poly­(3-hydroxybutyrate) (PHB) is a biocompatible and biodegradable polyhydroxyalkanoate, but its highly regular stereomicrostructure causes high crystallinity and poor processability. Here, we demonstrate that stereoirregular PHB can be produced microbially, challenging the view that strict PHA synthase stereospecificity makes such structures inaccessible. In a proof-of-concept study, we produced stereoirregular PHB containing 6.84% ± 0.04% (*S*)-3-hydroxybutyrate, with an *m*-dyad fraction of 11.8% ± 0.7%. Compared with conventional PHB, the stereoirregular material exhibited a reduced melting temperature, from 179.3 °C ± 0.7 to 154.3 °C ± 1.1 °C, and decreased molar-mass decomposition during processing. These results demonstrate that microbial synthesis can access stereochemically diverse PHB. Importantly, they show that biologically produced polymers can exhibit the improved processability and recyclability previously associated with chemically synthesized analogues. These findings overturn a long-standing assumption in PHB biosynthesis and provide a basis for biologically tuning polymer microstructure and performance through microbial engineering.

## Graphical abstract

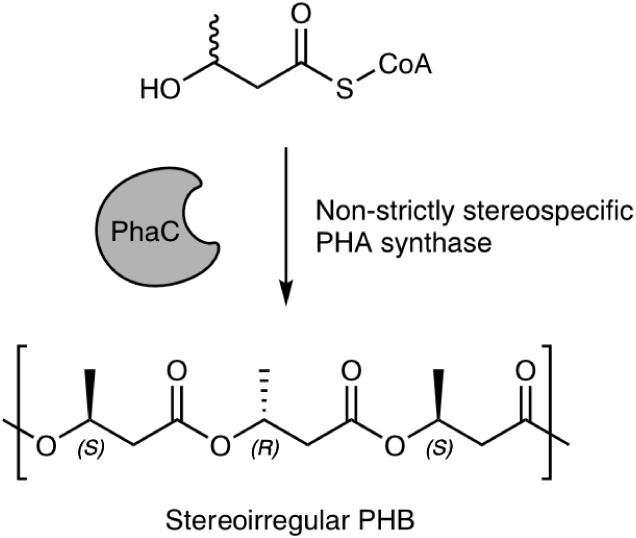

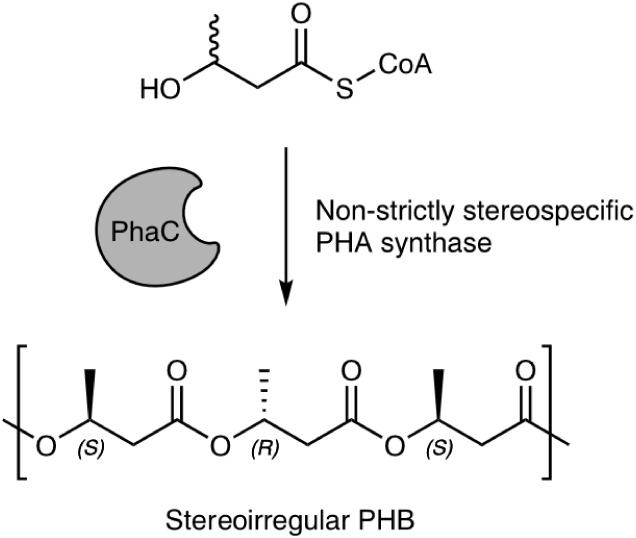

## 1 Introduction

Plastic production contributes to two major environmental challenges: persistent pollution due to the material’s resistance to degradation, and climate change because most plastics are derived from fossil resources.^1,2^ A promising biodegradable alternative to petroleum-based plastics is polyhydroxyalkanoates obtained from microorganisms, especially poly­(3-hydroxybutyrate) (PHB).^3,4^ However, several limitations have hindered its broader adoption.^4–6^ Natural, stereoregular (*R*)-PHB’s high crystallinity renders it brittle and raises its melting point to temperatures at which thermal degradation of the polymer chains occurs, complicating both processing and recycling.^7–9^

In polymer science, controlling stereochemistry is a well-established strategy to modulate crystallinity, thermal behavior, and mechanical performance. Accordingly, researchers have explored modifications to the typically isotactic stereochemistry of PHB by using chemical synthesis. The incorporation of the (*S*)-3-hydroxybutyrate monomer via chemical synthesis has been achieved to produce atactic (random) and syndiotactic (alternating) configurations.^10,11^ Increasing the fraction of the opposite enantiomer reduced both the crystallinity and melting temperature and improved material properties.^12,13^ However, these approaches are not currently competitive with microbial production, which remains the dominant route for PHB synthesis.^14–16^

Microbial synthesis of PHB offers several advantages: renewable resources (i.e., biomass, green hydrogen with CO_2_ or biogas) can be employed as feedstocks, and molar masses frequently outperform those obtained via chemical synthesis.^15,17–19^ However, the material properties of microbial PHBs remain a key drawback. Microorganisms exclusively produce (*R*)-isotactic PHB with the aforementioned shortcoming. PHB biosynthesis proceeds via a three-step metabolic pathway.^20^ Two molecules of acetyl coenzyme A (CoA) are condensed by acetyl CoA acetyltransferase (PhaA) to form acetoacetyl CoA, which is then reduced to (*R*)-3-hydroxybutyryl CoA, introducing the stereocenter and requiring NADPH as a cofactor (Figure 1, Supplementary Figure 1). Finally, PHA synthase (PhaC) polymerizes (*R*)-3-hydroxybutyryl CoA, releasing CoA. Because the stereocenter is not altered during polymerization, the resulting PHB consists exclusively of (*R*)-3-hydroxybutyrate monomers.^21^ To date, microbial synthesis of stereoirregular PHB has not been reported.

**Figure 1:**
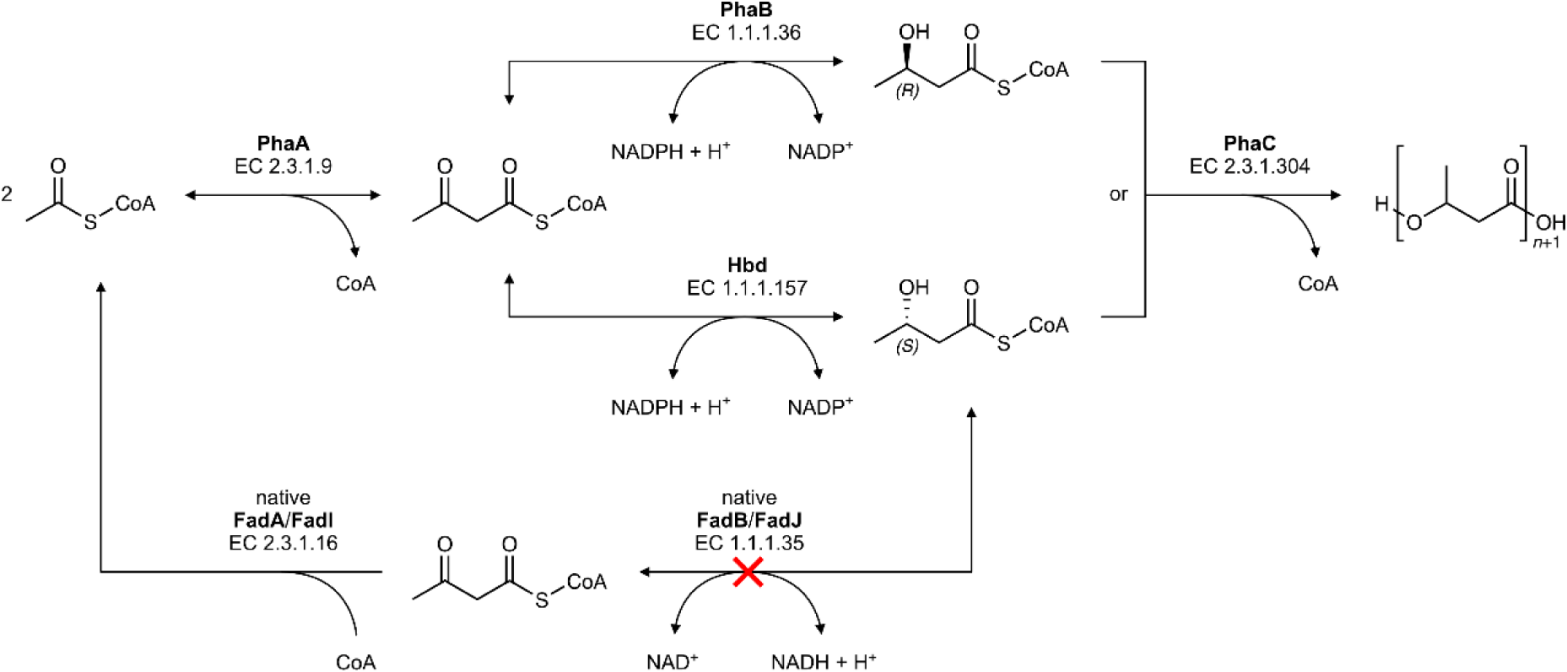
Metabolic pathway design for the synthesis of stereoirregular PHB in *Escherichia coli*. In addition to the PHB-synthesizing enzymes, PhaA, PhaB, and PhaC, Hbd is required to provide (*S*)-3-hydroxybutyryl CoA. Higher (*S*)-3-hydroxybutyrate fractions in the polymer can be obtained by deleting the homologs *fadB* and *fadJ*. The deletions prevent the degradation of (*S*)-3-hydroxybutyryl CoA to acetyl CoA via the fatty acid β-oxidation complex (FadA_2_B_2_/FadB_2_J_2_), which is selective for the (*S*)-enantiomer.

We hypothesized that the absence of stereoirregular PHB in biological systems may result from limited intracellular availability of (*S*)-3-hydroxybutyryl CoA rather than absolute exclusion by PHA synthases (Supplementary Description 1).^22–24^ With no evolutionary pressure on PHA synthases to exclude (*S*)-3-hydroxybutyryl CoA from polymerization, the longstanding belief that PHA synthases are strictly stereospecific for (*R*)-3-hydroxybutyryl CoA, therefore, might be a wrong assumption, considering that the stereocenter is introduced by acetoacetyl CoA reductase.^5,21,25–27^ If this were the case, microbial synthesis of stereoirregular PHB should be possible by providing (*S*)-3-hydroxybutyryl CoA intracellularly.

The present work is the first proof-of-concept attempting microbial production of stereoirregular PHB in recombinant *Escherichia coli*. This comprised the implementation of metabolic pathways for (*S*)-3-hydroxybutyryl CoA biosynthesis, identification of PHA synthases suitable of incorporating both enantiomers, characterization of these pathways, and fermentation scale-up to enable comprehensive analysis of the polymer’s structural, thermal, and mechanical properties.

## 2 Experimental Section

### 2.1 Media and Growth Conditions

Unless stated otherwise, *E. coli* was grown at 37 °C. Routine growth was performed in LB Miller medium (10 g L^–1^ sodium chloride, 10 g L^–1^ tryptone, 5 g L^–1^ yeast extract). PHB synthesis was performed in either LB Miller medium or MR minimal medium, both supplemented with 20 g L^–1^ glucose. MR medium (pH 6.9) contained 22 g L^–1^ potassium dihydrogen phosphate, 3 g L^–1^ diammonium hydrogen phosphate, 0.8 g L^–1^ citric acid, 0.7 g L^–1^ magnesium sulfate heptahydrate, and 5 mL L^–1^ trace metal solution.^28^ The trace metal solution was prepared in 0.5 M hydrochloric acid and consisted of 10 g L^–1^ iron sulfate heptahydrate, 2.7 g L^–1^ calcium chloride dihydrate, 2.2 g L^–1^ zinc sulfate heptahydrate, 1.0 g L^–1^ copper sulfate pentahydrate, 360 mg L^–1^ manganese sulfate monohydrate, 100 mg L^–1^ ammonium heptamolybdate tetrahydrate, and 20 mg L^–1^ sodium tetraborate decahydrate. Modifications to the MR medium (6.7 g L^–1^ potassium dihydrogen phosphate, 4.0 g L^–1^ diammonium hydrogen phosphate, 0.4 g L^–1^ magnesium sulfate heptahydrate) were made for fed-batch fermentation.^29^ For selection and maintenance of plasmids, media were supplemented with 100 μg mL^–1^ carbenicillin disodium salt, 34 μg mL^–1^ chloramphenicol, and 25 μg mL^–1^ kanamycin sulfate.

### 2.2 Strain Construction

Strains used in this study are listed in Supplementary Table 7. Production strains were derived from the Keio Collection of single-gene deletions.^30^ A kanamycin-sensitive strain was created from JW3822 by pCP20-mediated FLP recombination.^31^ The kanamycin-carrying deletion from JW2338 was picked up and transduced to the kanamycin-sensitive strain by P1 phage transduction.^32^ Another FLP recombination step yielded the kanamycin-sensitive double-deletion strain. Strains were verified by PCR amplification and Sanger sequencing of genomic loci.

### 2.3 Plasmid Construction

Plasmids used in this study are listed in Supplementary Table 8. Annotated sequences are available in Supplementary Data 1. Cloning was performed according to standard procedures.^33^ Gibson assembly was used to construct all plasmids unless stated otherwise. The antibiotic resistance of pUC19 was changed from ampicillin to kanamycin by replacing *bla* with *kan* amplified from pET-28a­(+). pUC-Kan-CnPhaCAB was created by amplifying the *phaCAB* operon from *Cupriavidus necator* H16 gDNA with 842 bp upstream and 568 bp downstream of the first and last gene, including the native σ70 promoter, and replacement of *lacZ* in pUC19-Kan in reverse orientation. Replacement of *phaC* to PHA synthases from other organisms was based on pUC-Kan-CnPhaCAB. *phaC* and *phaRC* from *Chromobacterium* sp. USM2 and *Priestia megaterium* 22–2, respectively, were synthesized. *phaC* STQK from *Pseudomonas* sp. 61–3 was subcloned from CBR_P_1189. *phaEC* was amplified from *Allochromatium vinosum* DSM 180 gDNA. pACYC184-CkHbd was created by replacing *tetR* with *hbd* from *Clostridium kluyveri* DSM 555 obtained via synthesis. Similarly, subcloning of superfolder GFP from pYTK047 gave pACYC184-sfGFP. The *hbd* sequence of pACYC184-CkHbd acted as a template for Hbd introduction into different positions in pUC-Kan-AvPhaEC-CnPhaAB and pUC-Kan-CsPhaC-CnPhaAB. pCkHbd was created by restriction–ligation cloning of the synthesized gene with NdeI/*Bam*HI into pET-28a­(+).

### 2.4 Recombinant Protein Production and Purification

Single colonies of transformed *E. coli* BL21­(DE3) were used to inoculate 100 mL baffled Erlenmeyer flasks containing 20 mL of LB medium supplemented with kanamycin sulfate (100 μg mL^–1^). Cultures were incubated overnight at 37 °C with shaking at 150 rpm. The following day, 2 L baffled Erlenmeyer flasks containing 400 mL of LB medium with the same antibiotic concentration were inoculated at a 1:100 ratio using the overnight cultures. These were grown at 37 °C with agitation at 100 rpm until the optical density at 600 nm (OD_600_) reached between 0.5 and 0.7.

Expression was initiated by adding IPTG to a final concentration of 1 mM, and the incubation temperature was lowered to 22 °C. Cells were collected the next morning by centrifugation at 8000*g* for 10 min. After discarding the supernatant, cell pellets were resuspended in 40 mL of binding buffer (20 mM sodium phosphate, pH 7.8; 150 mM sodium chloride; 20 mM imidazole). Cell lysis was performed twice for 10 min using a UIS250 V Ultrasonicator (Hielscher Ultrasonics) at 80% amplitude with 0.5-s pulses.

Lysate was clarified by centrifugation at 20,000*g* for 45 min at 4 °C. DNase I was added in small amounts, and the supernatant was filtered through a 0.2 μm syringe filter. His-tagged proteins were purified using His GraviTrap columns (Cytiva) following the manufacturer’s instructions, with elution carried out using buffer containing 20 mM sodium phosphate (pH 7.8), 150 mM sodium chloride, and 500 mM imidazole. Proteins were subsequently desalted into assay buffer (100 mM sodium phosphate, pH 7.8) using PD-10 desalting columns (Cytiva), also according to the manufacturer’s protocol.

The purified proteins were aliquoted and rapidly frozen in liquid nitrogen, then stored at −80 °C. Expression and purification success were confirmed by SDS-PAGE, and protein concentrations were measured at 280 nm using a NanoPhotometer P-Class (IMPLEN), based on their molecular weight and extinction coefficient.

### 2.5 Screening for PHB Operons Producing Stereoirregular PHB

*E. coli* BW25113 or *E. coli* BW25113 Δ*fadB* Δ*fadJ* was transformed with pUC-based and pACYC184-based plasmids and grown overnight. 100 mL baffled shake flasks containing 10 mL of prewarmed LB medium with 20 g L^–1^ glucose were inoculated with fresh colonies in triplicate. After 48 h of growth, cells were harvested by centrifugation, washed, freeze-dried, and the biomass was used for dry cell mass (DCM) and PHB quantification. Real cell mass (RCM) was defined as DCM subtracted by PHB mass.

### 2.6 Growth Rate Determination and Steady-State PHB Synthesis

Experiments were adapted from Tyo et al. (2010).^34^ *E. coli* BW25113 Δ*fadB* Δ*fadJ* was freshly transformed with the corresponding pUC-based plasmids and grown overnight. Single colonies were inoculated in triplicate into 14 mL cell culture tubes with 5 mL prewarmed LB medium and grown for 10 to 11 h. A volume of 0.2 mL was transferred to 14 mL cell culture tubes with 5 mL prewarmed MR medium and grown again for 10 to 11 h. Baffled 500 mL shake flasks with 50 mL prewarmed MR medium were inoculated to an OD_600_ = 0.02. Samples for OD_600_ measurements were taken at regular intervals, and the growth rate was determined after logarithmic transformation. When the exponentially growing cultures reached an OD_600_ = 2.0, samples for transcriptional profiling were taken and frozen at −80 °C. The remaining biomass was used for DCM and PHB quantification as described below.

### 2.7 Transcriptional Characterization of PHB Synthesis Operons

Transcriptional characterization was carried out as described in the literature.^34^ Briefly, RNA was extracted using the Monarch Total RNA Miniprep Kit (New England BioLabs), including an on-column DNase I digest of genomic and plasmid DNA. 60 ng of RNA was subjected to cDNA synthesis via the LunaScript RT SuperMix (New England BioLabs). 5 μL of a 100-fold dilution of the cDNA synthesis reaction was used as template for qPCR with the Luna Universal qPCR Master Mix (New England BioLabs). qPCR was performed on a CFX96 Touch Real-Time PCR Detection System (Bio-Rad). *phaA*, *phaB*, *phaC*, and *hbd* were quantified in relation to 16S rRNA (*rrsA*). Prior to relative quantification, sufficient amplification efficiency for each primer pair was validated using a dilution of PCR-amplified template. Primer specificity was verified by performing melt curve analysis after each run.

### 2.8 Dissolved Oxygen-Stat Fed-Batch Fermentation

Strains maintained as glycerol stocks were streaked on LB agar plates and grown at 37 °C for 24 h. A fresh colony was inoculated into a 14 mL cell culture tube containing 5 mL LB medium and grown at 37 °C for 8 h. 50 mL MR medium in a 500 mL baffled Schott shake flask was inoculated to an OD_600_ = 0.01 and grown at 30 °C for 24 h. The bioreactor, with an initial volume of 1.0 L MR medium, was inoculated to an OD_600_ of 0.1. Benchtop fermentation was carried out with a 2 L Univessel Glass cultivation vessel and Biostat B controller (Sartorius). Temperature was controlled at 30 °C. Dissolved oxygen was maintained at 30% by varying stirrer speed (500–1500 rpm) and constant air sparging (1.0 L min^–1^). PHB synthesis was induced upon oxygen limitation when the controller was unable to sustain the set point. pH was controlled on-sided at 6.80 by addition of 25% (v/v) ammonia–water. A dissolved oxygen-stat feeding strategy was employed, and a solution of 600 g L^–1^ glucose and 12 g L^–1^ magnesium sulfate heptahydrate was fed when dissolved oxygen rose to ≥45%. The feeding pulse added approximately 20 g of glucose. Foam suppression was achieved by automatic addition of a 10% Antifoam B emulsion (Sigma-Aldrich). Growth was monitored using a BioPAT Fundalux probe (Sartorius). Processes were stopped after dissolved oxygen started to rise. Data were evaluated with BioPAT MFCS 4.12.0 (Sartorius).

Approximately 10 mL samples were drawn from the reactor at regular intervals, from which OD_600_ was determined and clarified supernatant was obtained by centrifugation at 17,000*g* for 20 min. Supernatant samples were immediately stored at −20 °C until analysis. From the OD_600_ measurement, volumes equivalent to 20 mg biomass were washed twice (7000*g*, 5 min) with 10 mL of phosphate-buffered saline (pH 7.4) and transferred to a weighed vial for freeze-drying and GC-FID analysis.

### 2.9 PHB Extraction from Biomass

PHB was extracted from freeze-dried biomass by stirring one part biomass in 20 parts chloroform at 40 °C for 2 days. Cell debris was removed via a Büchner funnel, and the filtrate was dripped into two volumes of ice-cold ethanol. The precipitated PHB was separated by another filtration step and dried under vacuum.

### 2.10 Gas Chromatographic Analysis of PHB Stereocomposition

Weighted biomass or extracted PHB was resuspended in 0.5 mL of chloroform, and 0.25 mL of derivatization solution with the internal standard benzoic acid (85% methanol, 15% sulfuric acid, 0.7% (w/v) benzoic acid) was added. Derivatization to 3-hydroxybutyric acid methyl esters was performed at 95 °C for 3 h. After derivatization, samples were washed once with 0.5 mL double-distilled H_2_O, and the chloroform phase was used for measurement.

The enantiomeric composition of PHB was determined using a GC-2010 Plus gas chromatograph (Shimadzu). A 1 μL amount of the sample was introduced via split injection, with the injector maintained at 215 °C. Helium served as the carrier gas, flowing through a 25 m MEGA-DEX DAC-Beta column (MEGA) at a rate of 1 mL min^–1^ and a linear velocity of 28 cm s^–1^. Following injection, the oven temperature was ramped from 70 to 95 °C over a 5-min period, held at 95 °C for an additional 5 min, then increased to 215 °C over 12 min, where it was maintained for 3 min. The flame ionization detector was operated at a constant temperature of 250 °C. Signal integration was performed in GCsolution 2.42.00 (Shimadzu).

### 2.11 Ion Exclusion Chromatography of Carbohydrates and Organic Acids

Acetate, ethanol, formate, glucose, 3-hydroxybutyrate, lactate, pyruvate, and succinate were quantified using ion exclusion chromatography. Samples were thawed at room temperature and centrifuged at 17,000*g* for 20 minutes. The supernatant was diluted in 2.5 mM sulfuric acid, filtered through a 0.22 μm PVDF filter, and analyzed on an UltiMate 3000 system (Thermo Fisher Scientific) equipped with an UltiMate 3000 RS Diode Array Detector (Thermo Fisher Scientific) and an RI-101 refractive index detector (Shodex). 10 μL samples were injected into a flow of 0.5 mL min^–1^ 2.5 mM sulfuric acid on a Rezex ROA-Organic Acid H+ (8%) Ion Exclusion HPLC Column (Phenomenex) at 70 °C. Data analysis was performed in Chromeleon 6.80 (Thermo Fisher Scientific).

### 2.12 Analysis of Tacticity by Nuclear Magnetic Resonance Spectroscopy

Spectra were acquired using an Avance Neo 500 MHz NMR spectrometer (Bruker). Samples were dissolved in deuterated chloroform, and chemical shifts (δ) were reported relative to the solvent signal as an internal reference (^1^H: δ = 7.26 ppm, ^13^C: δ = 77.16 ppm). ^1^H NMR spectra were recorded with 16 scans and a 1.0 s delay, and ^13^C NMR spectra with 256 scans, a 2.0 s delay, and broadband ^1^H decoupling. The fraction of racemo diads (*f*_r_) was calculated from ^13^C spectra by fitting methyl group signals at 19.87 and 19.93 ppm after Gaussian apodization with a window size of 2.0 Hz and linear interpolation to 2^17^ data points: *f*_r_ = *A*_r_/(*A*_r_ + *A*_m_) × 100%, where *A*_r_ and *A*_m_ are the areas of racemo and meso diads, according to Huang et al.^19^ Data were processed using MestReNova 14.2.3 (Mestrelab Research). The expected *f*_r_ for a given (*S*)-3-hydroxybutyrate content (*f*_S_) was determined from fitting Monte Carlo simulation according to the formula *f*_r_ = 2­(*f*_S_ – 0.5)^2^ + 0.5.

### 2.13 Determination of Molar Mass Distribution by Size Exclusion Chromatography

PHB was dissolved in chloroform to a concentration of 5 mg mL^–1^ by gentle heating and subsequently filtered through a 0.2 μm PTFE filter. Analysis was performed on a SECcurity GPC System (Polymer Standards Service) with SDV columns (5 cm, 5 μm precolumn; 30 cm, 100,000 Å column; 30 cm, 1000 Å column; Agilent) and a 1260 Infinity refractive index detector (Agilent) at 23 °C. 50 μL samples were injected into a constant flow of 0.7 mL min^–1^ chloroform. Polystyrene standards from 1,250 g mol^–1^ to 1,670,000 g mol^–1^ were used for calibration. Data analysis was performed within PSS WinGPC UniChrom 8.33 (Polymer Standards Service).

### 2.14 Thermal Analysis

Melting and recrystallization behavior were analyzed by differential scanning calorimetry on a DSC 1 STAR System (Mettler Toledo). 10–20 mg samples were weighted into aluminum crucibles. Heating and cooling were performed twice from −30 to 200 °C at a rate of 10 K min^–1^ with 2-min isotherms and a nitrogen gas flow rate of 50 mL min^–1^. Crystallinity was calculated using the equation *X*_c_ = Δ*H*_m_/Δ*H* ^0^_m_ × 100%, where Δ*H*_m_ is the sum of measured melting enthalpies during heating and cooling, and Δ*H* ^0^_m_ is the melting enthalpy of PHB with 100% crystallinity in the ideal state. Δ*H* ^0^_m_ was given as 146 J g^–1^.^35^

Thermogravimetric analysis was used to analyze the thermal decomposition of PHB related to mass loss. 15–20 mg samples were measured under a constant air flow of 70 mL min^–1^ in a STA PT 1600 thermogravimeter (Linseis Messgeräte). Heating was performed at a rate of 5 °C min^–1^ until 100 °C, 10 °C min^–1^ until 600 °C, and 15 °C min^–1^ until 900 °C, which was held constant for 10 min. Aluminum oxide crucibles with lids were used for all measurements.

### 2.15 Production of Test Specimens and Tensile Testing

Approximately 0.6 g of PHB was compacted at 50 bar and room temperature in a cylindrical mold with a diameter of 2 cm. Unless noted otherwise, compacted isotactic and stereoirregular PHB was hot-pressed for 2.5 min into discs at 10 bar and 170 or 130 °C, respectively. Discs were left to age at 23 °C and 50% relative humidity for 1 week. Strips between 8 mm and 10 mm in width were cut from the material, their dimensions measured, and analyzed on a universal testing machine (smarTens 010, KARG Industrietechnik). Data were recorded and analyzed with LabMaster 2.5.11.3 (Hegewald & Peschke Mess- und Prüftechnik).

## 3 Results

### 3.1 Strain and Metabolic Pathway Design

We decided to synthesize stereoirregular PHB in recombinant *E. coli* because the organism is easy to alter genetically and delivers high intracellular PHB contents.^16,28,36^ To be able to synthesize stereoirregular PHB, *E. coli* requires the expression of four enzymes: PhaA and PhaB, which produce (*R*)-3-hydroxybutyryl CoA from acetyl CoA, an enzyme providing (*S*)-3-hydroxybutyryl CoA, and a suitable PhaC that is capable of polymerizing the mixture of 3-hydroxybutyryl CoA enantiomers (Figure 1). For the production of (*S*)-3-hydroxybutyryl CoA, we concluded that the enzyme class (*S*)-3-hydroxybutyryl CoA dehydrogenases (Hbds) would be the best choice. These enzymes perform a similar reaction to PhaB but with reverse enantioselectivity and NADH as their preferred cofactor. Under normal physiological conditions, the preference of NADH would drive the reaction in the undesired direction.^37^ Therefore, we selected Hbd from *Clostridium kluyveri* DSM 555, which strongly preferred NADPH to NADH and exclusively produced (*S*)-3-hydroxybutyryl CoA (Supplementary Table 1, Supplementary Figure 2).

Additionally, we rationally selected two gene knockouts that could elevate the fraction of (*S*)-3-hydroxybutyrate in the produced polymer. Our attention was drawn to the oxidation of acyl-CoAs to acetyl-CoAs by fatty acid β-oxidation (Figure 1, Supplementary Figure 1).^38^ We hypothesized that the complex could degrade (*S*)-3-hydroxybutyryl-CoA to acetyl-CoA. To prevent the futile cycle of (*S*)-3-hydroxybutyryl-CoA synthesis from acetyl-CoA via PhaA and Hbd and degradation via the complex, a Δ*fadB* Δ*fadJ* strain was constructed.

### 3.2 Identification of Non-Stereospecific PhaCs

The synthesis of stereoirregular PHB requires PhaCs that are able to polymerize a mixture of (*R*)- and (*S*)-3-hydroxybutyryl CoA. Out of the four PHA synthase classes, classes I, III, and IV are able to produce PHB. Five PhaCs were selected and screened for the synthesis of stereoirregular PHB: *Cupriavidus necator* H16 (class I, CnPhaC), *Chromobacterium* sp. USM2 (class I, CsPhaC), *Allochromatium vinosum* (class III, AvPhaEC), and *Priestia megaterium* 22–2 (class IV, PmPhaRC). The ability to produce stereoirregular PHB was also investigated for PhaC1 from *Pseudomonas* sp. 61–3 (PsPhaC1), as its STQK mutant is able to accumulate PHB despite being associated with class II.^39^ PHB synthesis was encoded by a *phaCAB* operon from *C. necator* on a high-copy-number vector controlled by its native σ70 promoter, and *phaC* from *C. necator* was replaced with the other *phaC* genes. *hbd* or superfolder *gfp* were constitutively expressed from a second low-copy-number vector with a different compatibility group.

The highest PHB contents could be obtained using CsPhaC and AvPhaEC (Figure 2, Supplementary Description 2). (*S*)-3-Hydroxybutyrate was only found in the produced polymers when Hbd was present. Surprisingly, PhaCs from all classes were able to produce (*S*)-3-hydroxybutyrate-containing PHB. The considerable variation in sequence similarity among the analyzed PhaCs (25%–59%, Supplementary Table 2) calls into question the commonly held belief in their strict stereospecificity. The strain deficient in β-oxidation showed significantly higher (*S*)-3-hydroxybutyrate fractions, demonstrating that degradation of (*S*)-3-hydroxybutyryl CoA was hindered (*p* < 0.05; two-tailed, two-sample, equal-variance *t*-test; except for PmPhaC and PsPhaC1, which had high variance due to very low PHB contents). As a result of the screening, we focused the remaining work on the double-deletion strain and on the PHA synthases CsPhaC and AvPhaEC.

**Figure 2:**
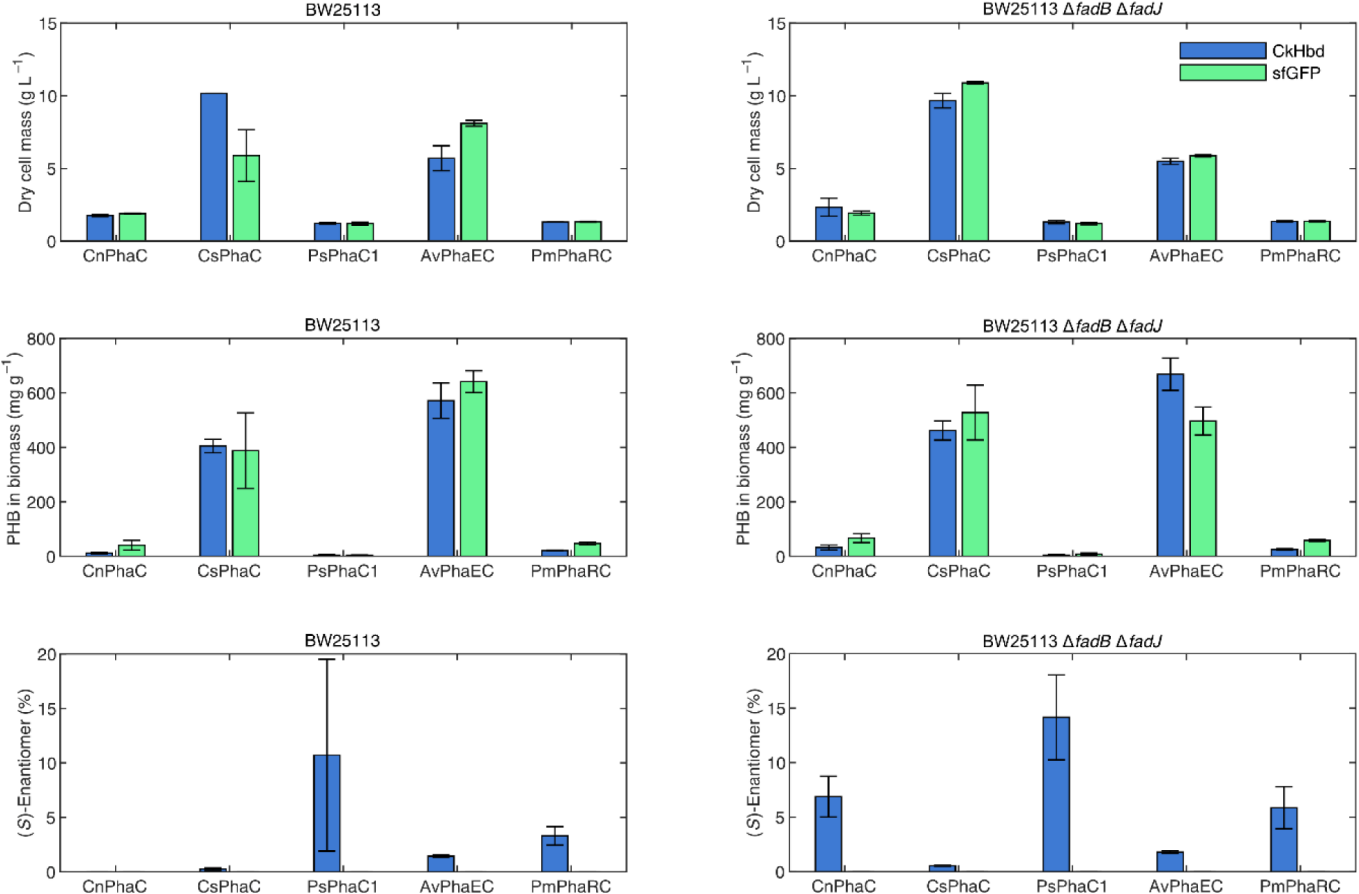
Screening for the incorporation of (*S*)-3-hydroxybutyrate into PHB by various PhaCs in a two-plasmid system in complex medium, expressing CkHbd or sfGFP as a control. The strain background was *E. coli* BW25113 with active or deficient fatty acid β-oxidation (Δ*fadB* Δ*fadJ*). The graphs show the mean and standard deviation of three biological replicates.

### 3.3 Optimization and Characterization of Metabolic Pathways

Next, we attempted to optimize the metabolic pathways by tuning expression levels. Rearranging genes in an operon was shown to be an excellent strategy for this purpose.^40^ Because it was evident that the gene order *phaCAB* is highly productive,^40^ not all possible variations were examined, and only the position of *hbd* was varied. Operons were constructed with CsPhaC or AvPhaEC and CkHbd expressed from three different positions (Supplementary Figure 3). Introducing the *hbd* gene into the operon led to much higher (*S*)-3-hydroxybutyrate fractions compared to the two-plasmid system (Figure 3). The highest fraction was obtained when *hbd* was placed at the second-last position of the operon. AvPhaEC was still the best producer of stereoirregular PHB, outcompeting CsPhaC, and reaching (*S*)-3-hydroxybutyrate fractions of 21% ± 5%, but at the cost of producing lower PHB amounts. Generally, there was a clear correlation between (*S*)-3-hydroxybutyrate fraction and PHB content. We assumed that this occurred because of (*S*)-3-hydroxybutyryl CoA accumulation, which limited free CoA available for the synthesis and polymerization of (*R*)-3-hydroxybutyryl CoA. Accumulation of (*S*)-3-hydroxybutyryl CoA could be caused by PHA synthases which still show strong stereopreference. Work from here onward was only carried out with AvPhaEC.

**Figure 3:**
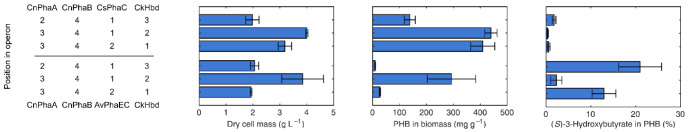
Effect of gene rearrangement on dry cell mass, PHB content, and (*S*)-3-hydroxybutyrate fraction in operons expressing CsPhaC or AvPhaEC and CkHbd in a one-plasmid system in complex medium. The graphs show the mean and standard deviation of three biological replicates.

The metabolic pathways arising from the different operon arrangements were characterized during steady-state growth so that the productivity of PHB synthesis was a measure of metabolic flux.^34^ To gain additional insights into how the metabolic pathways emerged, we determined transcript levels of the individual genes. Fitness of the strains, measured as growth rate, was similar or higher for the stereoirregular PHB-producing strains compared to the isotactic PHB-producing reference (strain A, Figure 4). Improved fitness in the strain expressing *hbd* at the center of the operon (strain C) could be attributed to lower metabolic burden, as indicated by lower PHB flux and (*S*)-3-hydroxybutyrate content. Somehow, rearrangement of the operon led to low overall expression of all genes and emerged as this phenotype. Again, the highest (*S*)-3-hydroxybutyrate fraction was observed with *hbd* at the second-last position (strain B) and at the cost of reducing PHB flux by one-third. Placement of *hbd* at this position also improved *phaB* expression, likely by enhancing transcription initiation (Supplementary Figure 4). We expected the highest (*S*)-enantiomer fractions with *hbd* furthest upstream in the operon (strain D), but it only showed intermediate flux and (*S*)-3-hydroxybutyrate fraction. The high expression of *hbd* could have overshadowed the expression of the other genes and therefore resulted in a less balanced metabolic pathway.

**Figure 4:**
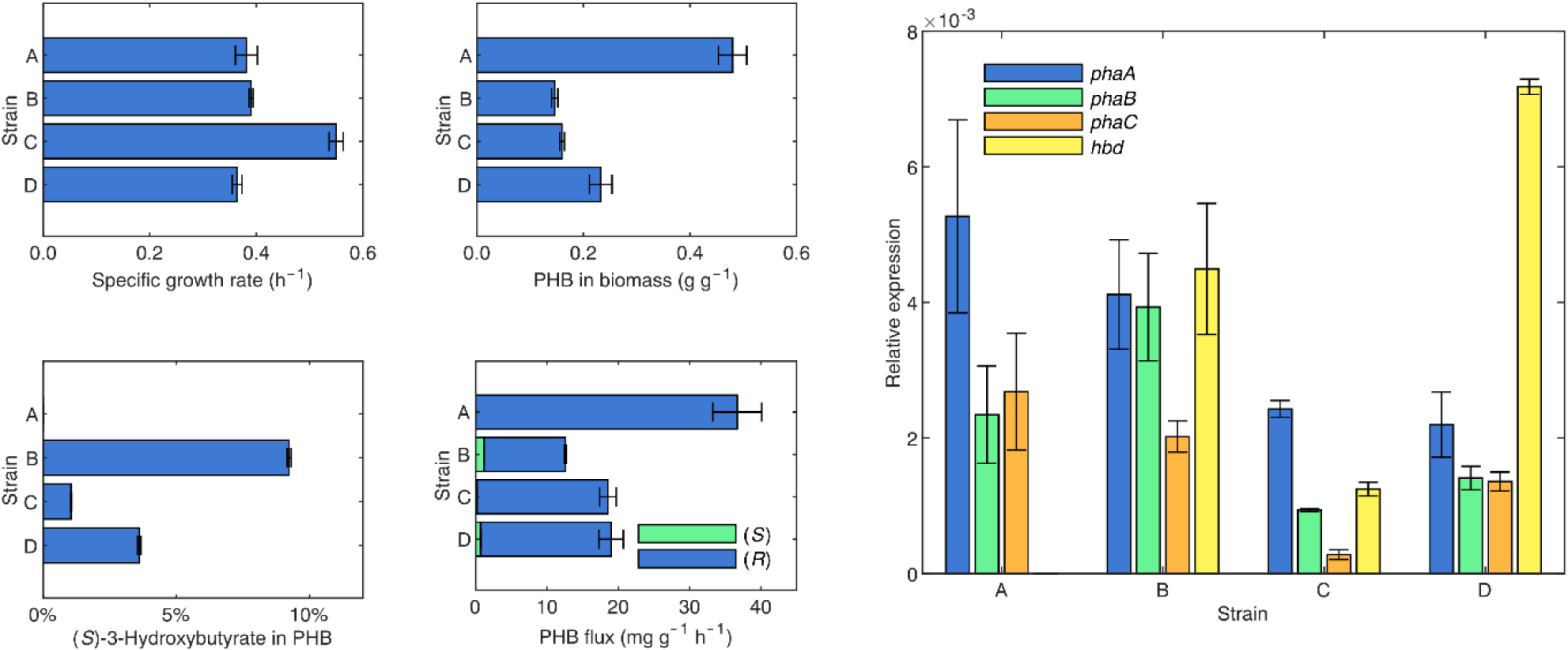
Characterization of metabolic pathways during steady-state growth in glucose minimal medium in a one-plasmid system arising from different gene arrangements in the operons. The graphs show the mean and standard deviation of three biological replicates. A, AvPhaEC-CnPhaAB; B, AvPhaEC-CnPhaA-CkHbd-CnPhaB; C, AvPhaEC-CkHbd-CnPhaAB; and D, CkHbd-AvPhaEC-CnPhaAB.

### 3.4 Upscaling and Production of Stereoirregular PHB

To obtain sufficient amounts of polymer for the analysis of its material properties, the synthesis of PHB was transferred to laboratory-scale bioreactors. A dissolved oxygen-controlled glucose fed-batch process in minimal medium was employed, which could be separated into two phases: a growth phase in which oxygen was abundant for biomass production, and an oxygen-limiting phase, which halted biomass growth and diverted carbon flux to the synthesis of PHB.^41^ Isotactic and stereoirregular PHB were produced by strains A and B, respectively (Supplementary 3).

Both strains had identical growth rates during the batch phase (Supplementary Table 3). With advancing time in the fed-batch phase, growth reduced in the stereoirregular PHB-producing strain. The fermentation of strain A could be clearly divided into a growth and a production phase, which was separated by oxygen limitation at 21 h, as evidenced by off-gas analysis (Figure 5a). Such a clear distinction between biomass accumulation and PHB synthesis could not be observed in strain B, despite oxygen limitation occurring at a similar time. After 25 h, the fermentation of strain B was stopped because no additional growth or PHB production occurred. Contrary to that, the fermentation of strain A was halted after the maximum volume of the culture vessel was reached, without any metabolic indicator that suggested PHB synthesis should diminish. This difference appeared in a reduction in PHB titer and productivity compared to the reference strain (Supplementary Table 3). While strain A had a similar PHB content compared to previous experiments ((0.443 ± 0.002) g g^–1^), a 3-fold reduction was observed in strain B ((0.046 ± 0.002) g g^–1^).

**Figure 5:**
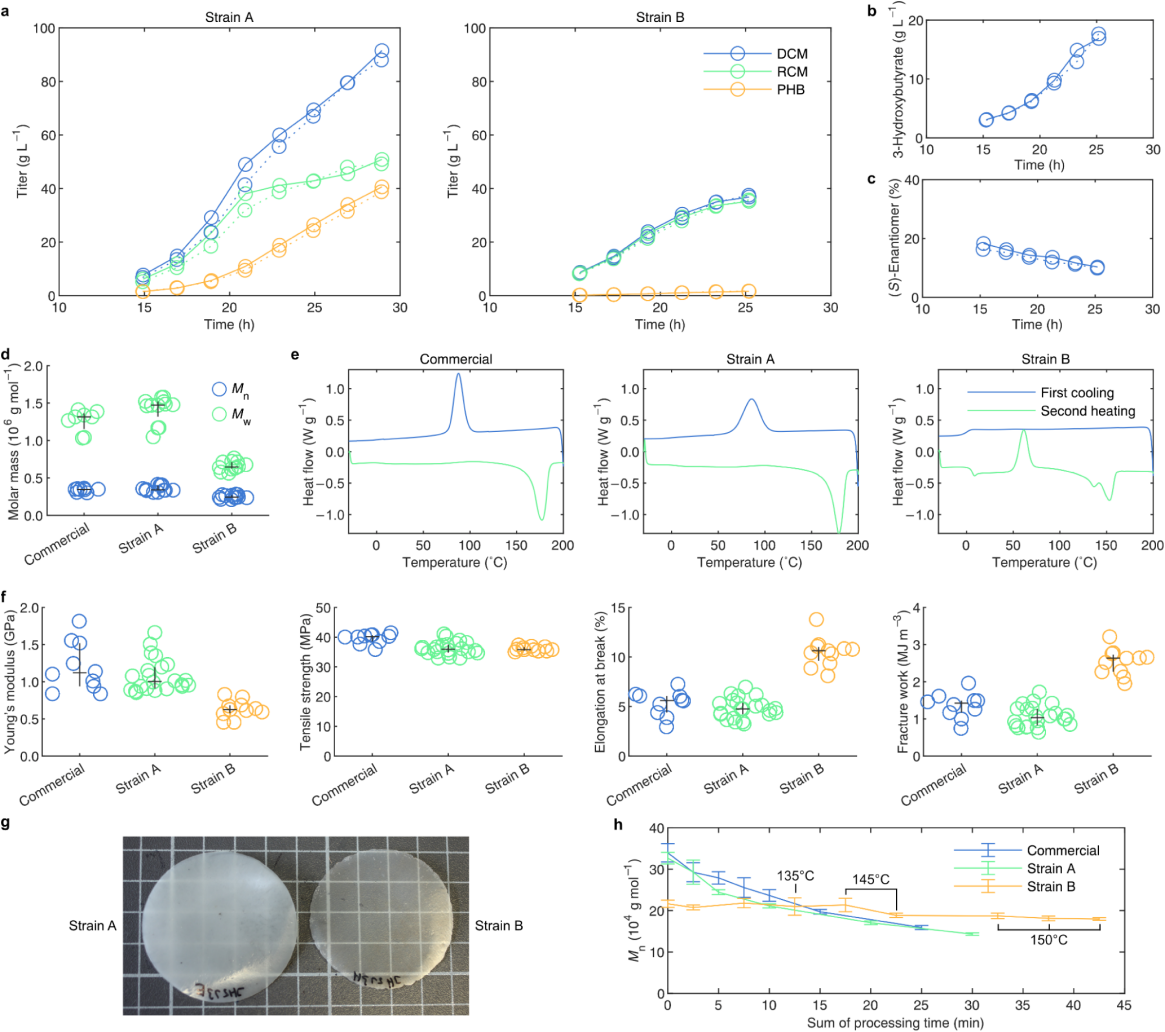
Synthesis and characterization of microbial PHB. (a) PHB synthesis by dissolved oxygen-controlled glucose fed-batch processes in minimal medium. Isotactic and stereoirregular PHB were produced by strains A and B, respectively. (b) Accumulation of extracellular 3-hydroxybutyrate by strain B. (c) (*S*)-3-Hydroxybutyrate content of PHB during fermentation of strain B. Molar mass distribution (d), thermal behavior during differential scanning calorimetry (e), and mechanical properties of tensile test specimens (f) of PHB. Images of processed PHB on a 1 cm grid (g) and molar mass decomposition of PHB during thermal processing (h). Isotactic and stereoirregular PHB were processed at 170 and 130 °C, unless shown otherwise. The graphs show biological duplicates in (a–c), representative measurements in (e), and median and interquartile range with all data points in (d) and (f), and mean and standard deviation of at least three material samples in (h). DCM, dry cell mass; RCM, real cell mass; *M*_n_, number-average molar mass; *M*_w_, weight-average molar mass.

To identify the cause of reduced growth and lower productivity in strain B, secreted metabolites were quantified using ion exclusion chromatography (Supplementary Figure 5). The two major mixed acid fermentation products that occurred during the fermentation of strain A were acetate and formate, both at a concentration below 5 g L^–1^. For strain B, the main product was acetate, which never reached concentrations higher than 4 g L^–1^ and, therefore, was unlikely to explain the growth defect. A strong time-dependent signal in the chromatograms of the fermentation of strain B was not present in the reference and could be identified as 3-hydroxybutyrate. At the end of the fermentation, strain B accumulated 17 g L^–1^ of 3-hydroxybutyrate, which proved to be almost exclusively made from the (*S*)-enantiomer (Figure 5b, Supplementary Figure 6). Previously, it was reported that *E. coli* contains native thioesterases that are capable of hydrolyzing 3-hydroxybutyryl-CoA.^24^ Therefore, it seemed that strain B counteracted the accumulation of (*S*)-3-hydroxybutyryl-CoA by hydrolysis, which released CoA to maintain metabolic activity. (*S*)-3-Hydroxybutyrate is then released as a byproduct into the culture medium via an unknown mechanism. Because strong acid secretion typically inhibits growth, this likely explains the growth reduction at higher cell densities.^42,43^

### 3.5 Structural, Thermal, and Mechanical Polymer Properties

Structural, thermal, and mechanical polymer properties were primarily compared between PHB synthesized under identical conditions (strains A and B), with commercial PHB used solely as a reference.

PHB was recovered from cells by chloroform extraction at an efficiency of around 80% (Supplementary Table 4). The extracted PHB proved to be free of detectable amounts of water, organic contaminants, salts, and antifoam, as evidenced by thermogravimetric analysis and nuclear magnetic resonance spectroscopy (Supplementary Figures 7–11).

The fraction of (*S*)-3-hydroxybutyrate monomers in the purified stereoirregular polymer was quantified to be 6.84% ± 0.04% (Supplementary Table 5). From ^13^C nuclear magnetic resonance spectra, the polymer microstructure was verified as isotactic-rich and stereoirregular (Supplementary Figure 11). The distribution of meso diads (11.8% ± 0.7%) proved to be close to the expected value of 12.7%, as determined by Monte Carlo simulation, emphasizing that the incorporation of (*S*)-3-hydroxybutyrate by PHA synthase is random.

The molar mass distribution of PHB samples was analyzed by size-exclusion chromatography (Figure 5d, Supplementary Table 5, Supplementary Figure 12). While the isotactic PHB showed similar distributions, the molar mass distribution of stereoirregular PHB was slightly shifted to shorter chain lengths but with a considerably less pronounced high-chain-length fraction. This was reflected in a lower molar mass dispersity (commercial, 4.1 ± 0.3; strain A, 4.4 ± 0.6; strain B, 2.8 ± 0.2), moderately lower number-average molar mass (commercial, (346 ± 23) kg mol^–1^; strain A, (369 ± 43) kg mol^–1^; strain B, (252 ± 25) kg mol^–1^) and a mass average molar mass reduced to less than half (commercial, (1402 ± 82) kg mol^–1^; strain A, (1590 ± 130) kg mol^–1^; strain B, (704 ± 64) kg mol^–1^). These results indicate that chain termination kinetics of PHA synthase are considerably altered when producing stereoirregular PHB. Whether this effect originated from (*S*)-3-hydroxybutyrate-incorporation-caused chain termination or lower productivity of strain B could not be concluded.

Thermal properties of the polymers were assessed via differential scanning calorimetry (Figure 5e, Supplementary Table 5). Similar to what was observed before,^44^ the stereoirregular polymer had two melting peaks. A reduction in melting temperature by around 20–25 °C was observed for the stereoirregular PHB at the second melt peak compared to its isotactic counterparts (commercial, 174.7 °C ± 1.3 °C; strain A, 179.3 °C ± 0.7 °C; strain B, 154.3 °C ± 1.1 °C). Assuming equal specific enthalpies of fusion, degrees of crystallinity were calculated to be 54.7% ± 0.7% for commercial PHB, 55.0% ± 1.7% for strain A, and 39.1% ± 3.2% for strain B.

The bulk of industrial PHB processing is performed via thermal techniques—mainly extrusion and injection molding. Because thermal history can influence material properties, samples were treated identically to allow comparability. Comparable amounts of PHB were compacted and hot-pressed into discs. With similar pressure and time, equivalent thickness and diameter of the discs could be produced at 170 °C for isotactic and at 130 °C for stereoirregular PHB. At a material thickness of around 0.2–0.3 mm, the stereoirregular PHB appeared visually transparent, whereas isotactic PHB was opaque (Figure 5g).

Young’s modulus of the stereoirregular polymer compared to its isotactic counterpart was approximately half (Figure 5f, Supplementary Table 5, Supplementary Figure 13; commercial, (1.2 ± 0.3) GPa; strain A, (1.1 ± 0.2) GPa; strain B, (0.6 ± 0.1) GPa), while elongation at break slightly improved (commercial, 5.3% ± 1.3%; strain A, 4.8% ± 1.0%; strain B, 10.5% ± 1.5%) without compromising tensile strength. The mechanical properties of microbial, stereoirregular PHB were summarized by a higher fracture work (commercial, (1.4 ± 0.4) MJ m^–3^; strain A, (1.1 ± 0.3) MJ m^–3^; strain B, (2.5 ± 0.4) MJ m^–3^).

Molar mass decomposition of PHB begins at temperatures between 175 and 180 °C. Since the melting temperature of stereoirregular PHB was significantly lower, we investigated how repeated processing cycles affected molar mass. Processing was performed at temperatures comparable to those used for the production of tensile test specimens. A pronounced reduction in molar mass was observed in both isotactic polymers; number-average molar mass decreased by 53% over 25 min and by 56% over 30 min at 170 °C for commercial isotactic PHB and isotactic PHB from strain A, respectively (Figure 5h). In contrast, the decomposition of stereoirregular PHB was significantly lower (17% reduction in number-average molar mass over 42.5 min) with prolonged processing time, despite a stepwise increase of processing temperature to 150 °C (Supplementary Description 5, Supplementary Figure 13). Although isotactic PHB initially has a favorable molar mass distribution, prolonged heat treatment diminished its advantage below the molar mass of stereoirregular PHB.

## 4 Discussion

For the design of the microbial production strain, we argued that metabolism must meet two requirements to be capable of producing stereoirregular PHB. The first requirement was the availability of (*S*)-3-hydroxybutyryl-CoA, which was successfully achieved by supplying the enzyme (*S*)-3-hydroxybutyryl-CoA dehydrogenase. Second, a suitable PHA synthase capable of polymerizing both (*R*)- and (*S*)-3-hydroxybutyryl-CoA was needed. From our data, it can be concluded that a broad range of PHA synthases can perform these reactions.

Nonetheless, we observed multiple limitations, suggesting that PHA synthases are not yet the perfect catalyst for stereoirregular PHB production. Comparing activity data of the two enzymes producing (*R*)- and (*S*)-3-hydroxybutyryl CoA shows that the (*S*)-enantiomer-creating reaction is considerably faster (3.14-fold; PhaB, *v*_max_ = (162 ± 6) μmol min^–1^ mg^–1^; Hbd, *v*_max_ = (509 ± 6) μmol min^–1^ mg^–1^).^45^ In the context of the transcription data, this suggests that cells are very likely flooded with (*S*)-3-hydroxybutyryl CoA leaving little CoA available for the synthesis of (*R*)-3-hydroxybutyryl CoA. Initially, growth dilution seems to be sufficient to remove (*S*)-3-hydroxybutyryl CoA, but when growth rate decreased, cells could only fall back to detoxification by (*S*)-3-hydroxybutyrate secretion. This mechanism probably explains the strong decline in PHB productivity in the production phase. Despite the high (*S*)-3-hydroxybutyryl CoA abundance, only moderate amounts of (*S*)-3-hydroxybutyrate were found in the produced polymer, emphasizing that PHA synthase is the bottleneck of stereoirregular PHB synthesis. As previously argued, this likely originates in the PHA synthase’s overall but not strict preference of (*R*)- over (*S*)-3-hydroxybutyryl CoA. Identifying or engineering a less selective PHA synthase would probably solve this problem. With such a biocatalyst, stereoirregular PHB with similar properties should be obtained with much higher productivity and titer at lower (*S*)-3-hydroxybutyryl CoA dehydrogenase levels. Consequently, the limitation of this work provides a clear roadmap for future work.

One advantage of stereoirregular PHB is that both monomers, (*R*)- and (*S*)-3-hydroxybutyrate, are synthesized from the same metabolic precursors, simplifying strain design and process control because a single carbon source can be used. More often than not, the synthesis of polyhydroxyalkanoate copolymers involves two metabolic pathways^46,47^ and therefore at least two carbon sources, resulting in more complex and challenging metabolism.^48^

Earlier works had assessed the structure–property correlations expected from PHB with mixed-stereoisomer chain structures by assessing synthetic materials with varying ratios of (*R*)- and (*S*)-enantiomers (Supplementary Table 6).^12,13,49,50^ Having determined that the molecular structure of biosynthesized stereoirregular PHB corresponds to that obtained by syntheses with a random catalyst,^12^ we discuss below to what extent the thermal and mechanical properties—most relevant for processing and application—were effectively altered.

Arguably, the single strongest influence on the processability and utility of a given polymer is its tendency to form stable crystals under given conditions. Simply put, these constitute mechanical anchors that counteract plastic deformation. As pointed out in the introduction and in the literature,^51^ it is a typical aim of studies on chemical modification, copolymerization, or addition of PHB to generally reduce the amount of crystallization. We hypothesized that it would be possible to directly produce PHB with properties that would overcome the known obstacles to its wider utility. On the basis of the sum of observations, this hypothesis can be answered in the affirmative:

Under the same conditions, biosynthesized stereoirregular PHB showed a reduced total enthalpy of fusion. Earlier studies found that lowered total enthalpies of fusion in PHB with increasing stereoirregularities indicate not only a smaller total fraction of the crystalline phase but also an increase in its structural disturbance.^52^ Concurrently—and crucial for processing—the melting point depression also observed in the literature^13,50^ was confirmed and accompanied by a broader melt transition signal. Taken together, these observations indicate an increased nucleation activation energy and, therefore, a reduced expected crystallization rate according to Lauritzen–Hoffman theory.^13^ This was confirmed by the distinct cold crystallization exotherm observed upon heating the stereoirregular PHB, but not the purely isotactic species, where crystallization was completed during the cooling cycle.

We expected the reduction in the proportion of crystalline regions to lead to a greater facility for plastic deformation. Indeed, under the same conditions, the elongations at break of biosynthesized stereoirregular PHB were significantly higher than those of isotactic PHB. Notably, the tensile strength remained similar for all types of materials. This increased ductility also translated to larger works of fracture in biosynthesized stereoirregular PHB. It should be noted that tensile testing was carried out on films, which were found to yield properties that may differ from those obtained from bulk materials.^35,50^

To relate the thermoforming properties of biosynthesized stereoirregular PHB to those of purely isotactic PHB, we compared the temperatures required to form equivalent amounts of PHB. Considering only nucleation, the increased activation energy in stereoirregular PHB can be expected to allow plastic deformation at lower temperatures, which is in agreement with empirical findings.^53^

Additionally, our findings indicate that stereoirregular PHB can be processed with fewer compromises with regard to its material properties, as molar mass decomposition is strongly reduced because of the shift in melting temperature. Moreover, they suggest that stereoirregular PHB may be recycled more frequently than currently possible for isotactic PHB before the deterioration of its mechanical properties.

The stereochemical composition of our PHB suggests it may be more readily degradable than the fully isotactic polymer. Kemnitzer et al.^49^ showed that higher fractions of the (*S*)-enantiomer reduce crystallinity and improve accessibility to PHA depolymerases, with biodegradability increasing up to about a 20% (*S*)-enantiomer fraction before additional amounts impede enzymatic attack. Based on the established relationship, the 6.84% (*S*)-enantiomer fraction in the microbial stereoirregular PHB probably enhances its susceptibility to degradation.

## 5 Conclusions

The material properties of isotactic PHB are insufficient for broad industrial application because of its high degree of crystallinity, which results in brittleness and molecular-weight decomposition at processing temperatures. Chemical synthesis has shown that these problems can be overcome by producing a stereoirregular polymer. Currently, chemical synthesis is not the industrial route of PHB synthesis—PHB is produced microbially. The present proof-of-concept has addressed this gap and, for the first time, provides a microbial synthesis route for stereoirregular PHB, which showed the improved material properties and processability/recyclability hinted at by chemical synthesis.^12^

This work suggests that biological systems, known and often appreciated for their high stereospecificity, can be engineered to access stereochemical diversity. In particular, the generation of defined monomer pools by metabolic engineering offers a complementary approach to polymer design, thereby enabling PHB synthesis under mild conditions from renewable feedstocks. Extending this concept beyond PHB to other polyhydroxyalkanoates or enzymatic polymerization systems could enable broader control over polymer structure–property relationships in sustainable materials.

## Supplementary Material





## Notes

Supporting Information: detailed descriptions of metabolism, process control, and polymer processing; specific enzyme activities, stereoselectivity, and sequence similarity; gene arrangement in operons and GC content; process metrics, fermentation byproducts, and polymer recovery efficiency; thermogravimetric analysis, nuclear magnetic resonance spectra, size exclusion chromatography traces, and tensile testing curves; summary of structural, thermal, and mechanical properties; comparison of properties with the literature; strains and plasmids (PDF)
Supplementary Data: annotated plasmid maps (ZIP)
