# Supplementary material for: Engineering Access to Stereoirregular Polymer Microstructures Enables Improved Processability of Microbial Poly(3-hydroxybutyrate)
Source: Biomacromolecules. 2026 Jun 12;27(7):4898–908. doi: 10.1021/acs.biomac.6c00971 (PMC13370766; doi:10.1021/acs.biomac.6c00971)
Supplement: Supplementary file 1 [file bm6c00971_si_001.pdf]

Supporting Information

Engineering Access to Stereoirregular Polymer Microstructures Enables Improved Processability of Microbial Poly(3-Hydroxybutyrate)

Marcel Mayer,[1,3,4] Julian Helberg,[2] Kai Stirnweiß,[1] Navaneeth Shiva Kumar,[1] Daniel Van Opdenbosch,[2] Doris Schieder,[1] Cordt Zollfrank[2,4] & Volker Sieber[1,3,4,5,*]

1 Chair of Chemistry of Biogenic Resources, Campus Straubing for Biotechnology and Sustainability, Technical University of Munich, 94315 Straubing, Germany

2 Chair of Biogenic Polymers, Campus Straubing for Biotechnology and Sustainability, Technical University of Munich, 94315 Straubing, Germany

3 SynBioFoundry@TUM, Technical University of Munich, 94315 Straubing, Germany

4 Center for Microplastic Prevention, Technical University of Munich, 94315 Straubing, Germany

5 Catalysis Research Center, Technical University of Munich, 85748 Garching, Germany

* Corresponding author, Volker Sieber, sieber@tum.de

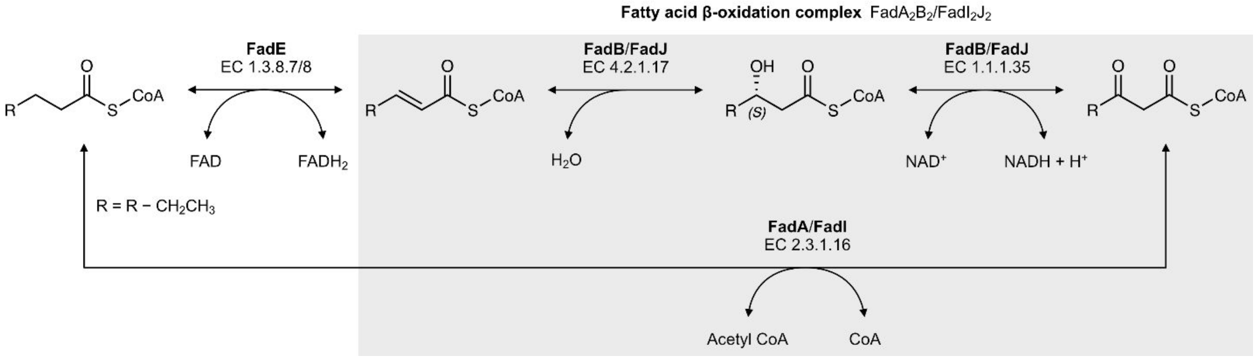

Supplementary Figure 1: Reactions of fatty acid degradation by the fatty acid β-oxidation complex $FadA_2B_2/FadI_2J_2$. The two homologs FadB and FadJ perform hydration of *trans*-Δ^2-enoyl CoA to (*S*)-3-hydroxyacyl CoA and oxidation of (*S*)-3-hydroxacyl CoA to 3-ketoacyl CoA. Because the complex runs iteratively, shortening the alkyl chain by two carbon units, (*S*)-3-hydroxybutyryl CoA should also be a substrate, which is degraded to two acetyl CoA molecules. The synthesis of stereoirregular PHB yields (*S*)-3-hydroxybutyryl CoA from acetyl CoA, which then can be degraded to acetyl CoA by the complex. Deleting FadB and FadJ prevents this futile cycle.

Supplementary Description 1: Availability of (*S*)-3-hydroxybutyryl CoA intracellularly.

To the best of our knowledge, quantification of 3-hydroxybutyryl CoA has not been attempted in non-PHB-producing *Escherichia coli*. As a result, we tried to deduce the probability of its presence from available data. A search for pathways in the EcoCyc database[1] for reactions where the compound *(S)-3-hydroxybutanoyl-CoA* participates gave two results: *oleate β-oxidation* and *fatty acid β-oxidation I (generic)*. Involvement of (*S*)-3-hydroxybutyryl CoA in the two pathways is due to the same reaction catalyzed by the fatty acid β-oxidation complex encoded by the operons *fadBA* and *fadIJ*. The complex performs hydration, oxidation, and thiolysis of *trans*-Δ^2-enoyl-CoA in three successive steps (Supplementary Figure 1). (*S*)-3-Hydroxybutyryl CoA is non-covalently bound to the complex via its adenine moiety and only the fatty acid tail is shuttled between the hydration and oxidation reactions.[2] Because (*S*)-3-hydroxybutyryl CoA is only an intermediate in the reaction sequence, it seems unlikely that considerable amounts are released from the complex into the cytoplasm. We therefore assume that the presence of free (*S*)-3-hydroxybutyryl CoA is negligible in wildtype organisms. Nonetheless, it seems probable that (*S*)-3-hydroxybutyryl CoA could enter the complex, bind via its adenine moiety, and becomes oxidized if available in the cytoplasm.

Supplementary Table 1: Specific activity of Hbd enzymes from *Clostridium acetobutylicum* and *Clostridium kluyveri* for acetoacetyl CoA reduction with NADH or NADPH at pH 7.8 and 30°C. Values are mean and standard deviation of three biological replicates.

Enzyme	v_{NADH} (µmol min^{-1} mg^{-1})	v_{NADPH} (µmol min^{-1} mg^{-1})
CaHbd	673 ± 37	8.9 ± 0.9
CkHbd	4.6 ± 0.2	509 ± 6

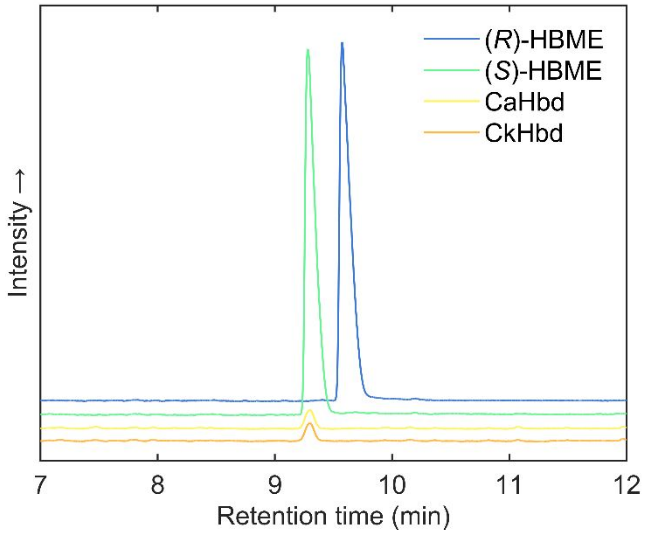

Supplementary Figure 2: Chirality of derivatized 3-hydroxybutyryl CoA produced by CaHbd and CkHbd after full conversion as determined by GC-FID and pure (*R*)- and (*S*)-HBME as reference. (*R*)-HBME, (*R*)-3-hydroxybutyric acid methyl ester; (*S*)-HBME, (*S*)-3-hydroxybutyric acid methyl ester

Supplementary Description 2: Error sources of in vivo PHB content analysis via GC-FID.

Quantification of PHB by GC-FID can be impacted by 3-hydroxybutyrate, free or bound as esters, as long as it can be derivatized to 3-hydroxybutyric acid methyl ester during sample preparation. Both extracellular and intracellular sources can contaminate quantification. To prevent contamination from extracellular sources, cells were carefully washed before lyophilization. One should be aware that intracellular sources other than PHB could have had an impact on quantification too. Intracellular concentration of 3-hydroxybutyryl coenzyme A were quantified by [3] and are a negligible error source even compared to low intracellular PHB amounts. Concentrations of intracellular 3-hydroxybutyrate have not been determined in PHB producing microorganisms that are unable to depolymerize PHB. Therefore, we could not rule out that PHB quantification by GC-FID was distorted by 3-hydroxybutyrate. Because screening for PHA synthases was aimed at high PHB contents and (*S*)-3-hydroxybutyrate fractions, we believe GC-FID quantification offers a convenient tradeoff between accuracy and speed until the final production strain was established to produce sufficient PHB for unbiased analysis of the purified polymer.

Supplementary Table 2: Pairwise amino acid sequence alignment of PhaCs.

	CnPhaC	CsPhaC	PsPhaC1	AvPhaC	PmPhaC
CnPhaC	100.0%	58.8%	48.9%	27.5%	26.7%
CsPhaC		100.0%	50.6%	25.9%	27.6%
PsPhaC1			100.0%	28.7%	24.7%
AvPhaEC				100.0%	55.0%
PmPhaC					100.0%

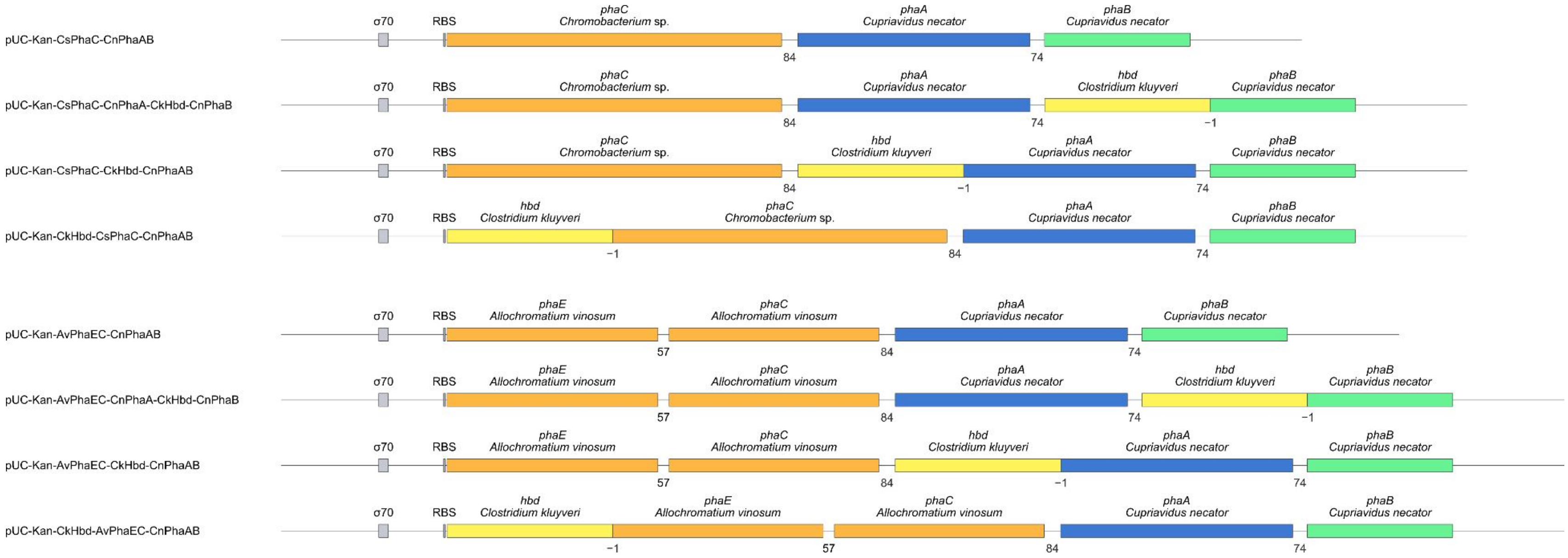

Supplementary Figure 3: Arrangement of genes in synthetic operons for plasmids with CsPhaC and AvPhaEC. The nucleotide distance between stop codon and start codon is indicated by numbers below each scheme.

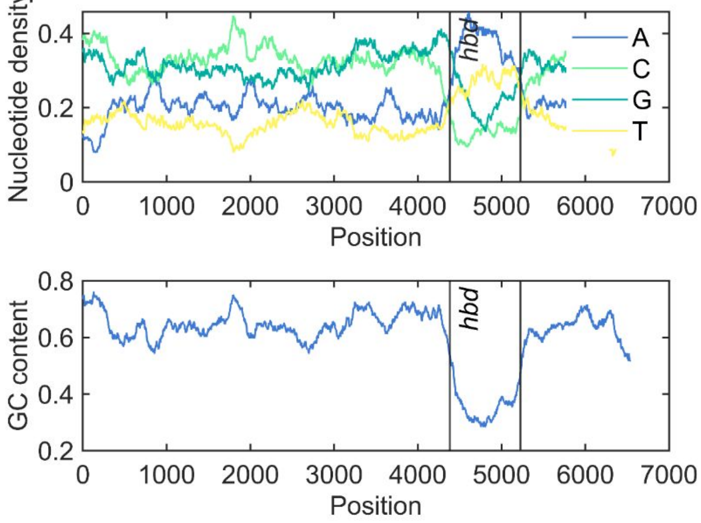

Supplementary Figure 4: The low GC content of the *hbd* gene could enhance DNA melting by RNA polymerase compared to the high GC content of the operon. GC content was calculated over a window of 200 nucleotides. The operon depicted is AvPhaEC-CnPhaA-CkHbd-CnPhaB from strain B.

Supplementary Description 3: Feed control differences between the PHB production processes.

The feed control of the fermentations producing isotactic and stereoirregular PHB could not be performed identical because the precipitation of insoluble struvite (ammonium magnesium phosphate hexahydrate) clogged the air supply to the culture vessels. Both processes were run as dissolved oxygen fed-batch processes but the process producing stereoirregular PHB, required a large feed pulse at 9.5 h to prevent struvite formation during the later phases of the process. After 10 h, the feed pulse added approximately 60 g glucose and 1.2 g magnesium sulfate heptahydrate to the culture broth, which likely increased overflow metabolism and, as a result, decreased growth rate slightly. However, we think that it was highly implausible that the lower PHB titer and higher 3-hydroxybutyrate secretion was caused by the differences in process control rather than the distinct metabolisms. Feed control during the production phase was identical in both processes.

Supplementary Table 3: Process metrics for the production of isotactic and stereoirregular PHB. Data are given for biological duplicates. RCM, real cell mass; DCM, dry cell mass

Metric	Strain A		Strain B	
Process time (h)	28.90	28.93	25.18	25.20
Batch growth rate (h^{-1})	0.35	0.34	0.36	0.36
Doubling time (min)	117	122	116	116
PHB titer ($g\ L^{-1}$)	38.8	40.6	1.76	1.61
RCM titer ($g\ L^{-1}$)	49.2	50.9	35.7	35.2
DCM titer ($g\ L^{-1}$)	88.0	91.5	37.5	36.8
PHB productivity ($g\ L^{-1}\ h^{-1}$)	1.34	1.40	0.07	0.06
RCM productivity ($g\ L^{-1}\ h^{-1}$)	1.70	1.76	1.42	1.40
DCM productivity ($g\ L^{-1}\ h^{-1}$)	3.04	3.16	1.49	1.46
PHB yield ($g\ g^{-1}$)	0.20	0.20	0.012	0.011
RCM yield ($g\ g^{-1}$)	0.25	0.25	0.24	0.23
DCM yield ($g\ g^{-1}$)	0.45	0.45	0.25	0.25
PHB content (%)	44.1	44.4	4.7	4.4

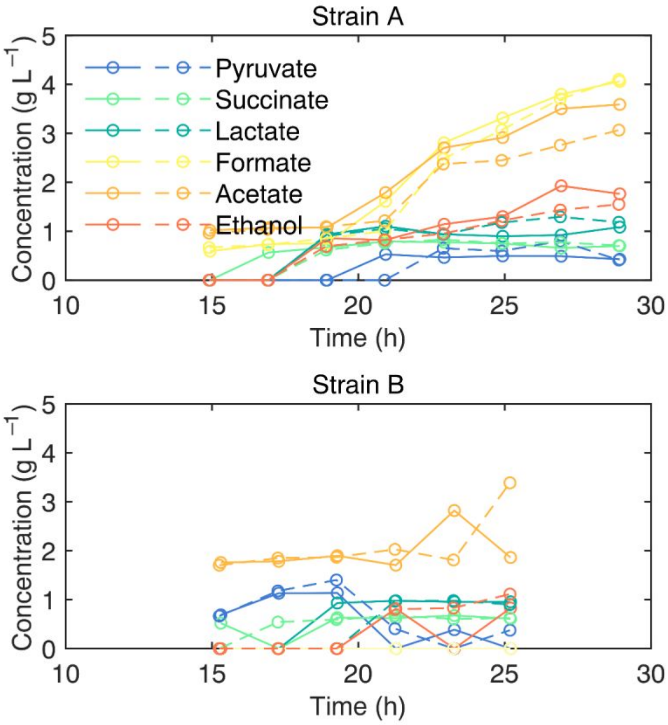

Supplementary Figure 5: Analysis of mixed acid fermentation products during the production of isotactic and stereoirregular PHB via strains A and B. The graphs show biological duplicates.

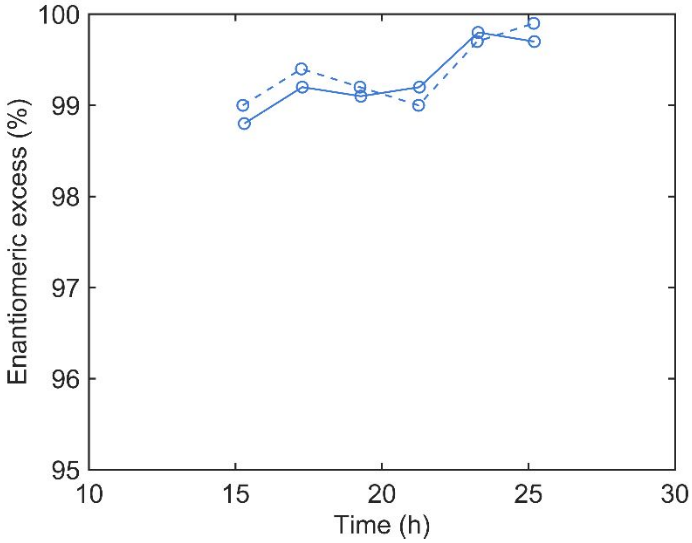

Supplementary Figure 6: Enantiomeric excess of of (*S*)-3-hydroxybutyrate secreted into the medium during stereoirregular PHB production. The graph shows biological duplicates.

Supplementary Table 4: PHB recovery efficiency from biomass through chloroform extraction. Data are given for biological duplicates. DCM, dry cell mass

	Strain A		Strain B	
Recovered DCM (g)	120.2	122.2	52.8	49.2
Intracellular PHB (g)	53.0	54.2	2.48	2.15
Extracted PHB (g)	44.2	42.7	2.11	1.67
Efficiency (%)	83	79	85	78

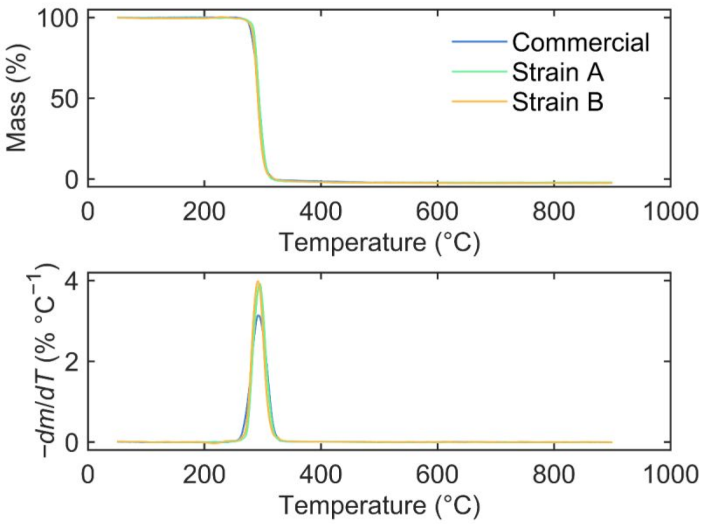

Supplementary Figure 7: Thermogravimetric analysis of PHB samples.

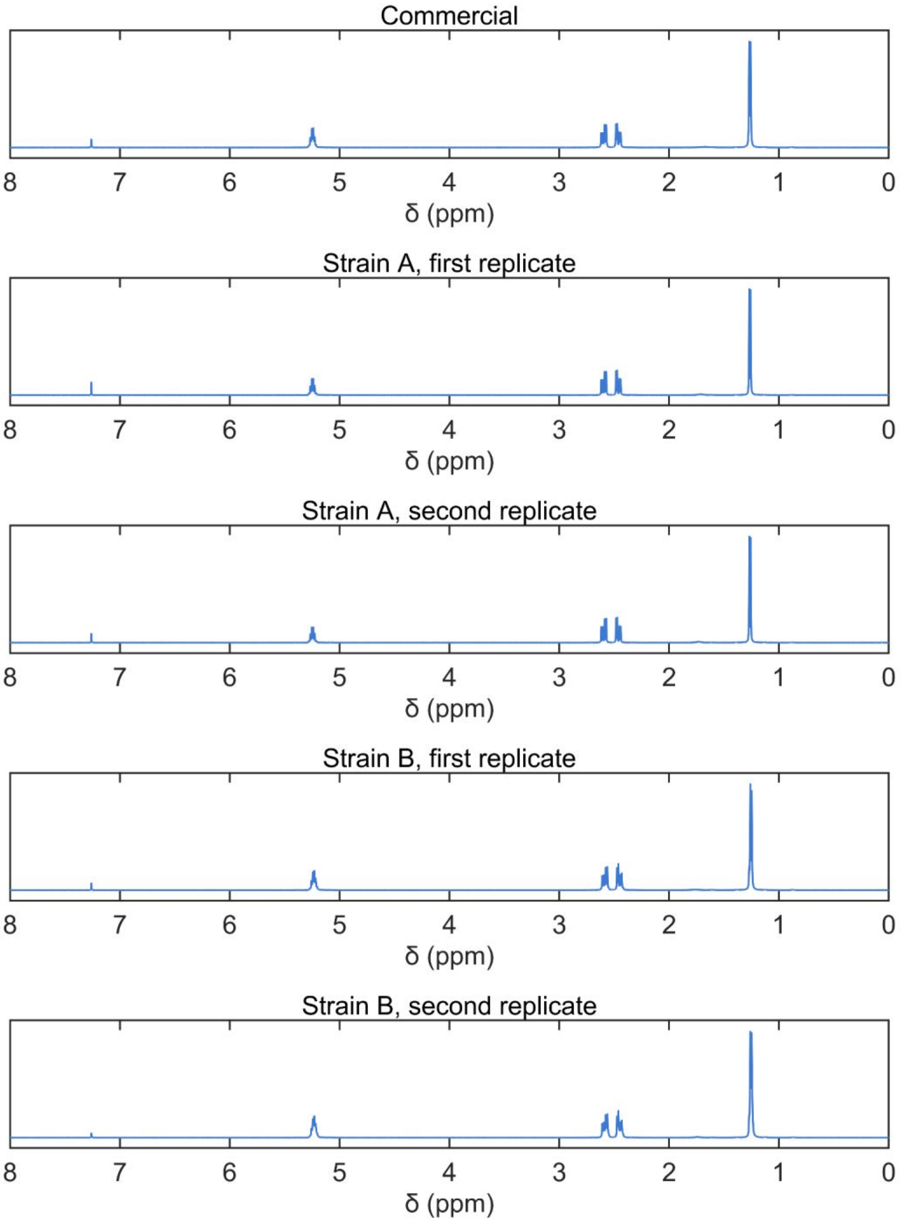

Supplementary Figure 8: ^{1}H NMR spectra of PHB.

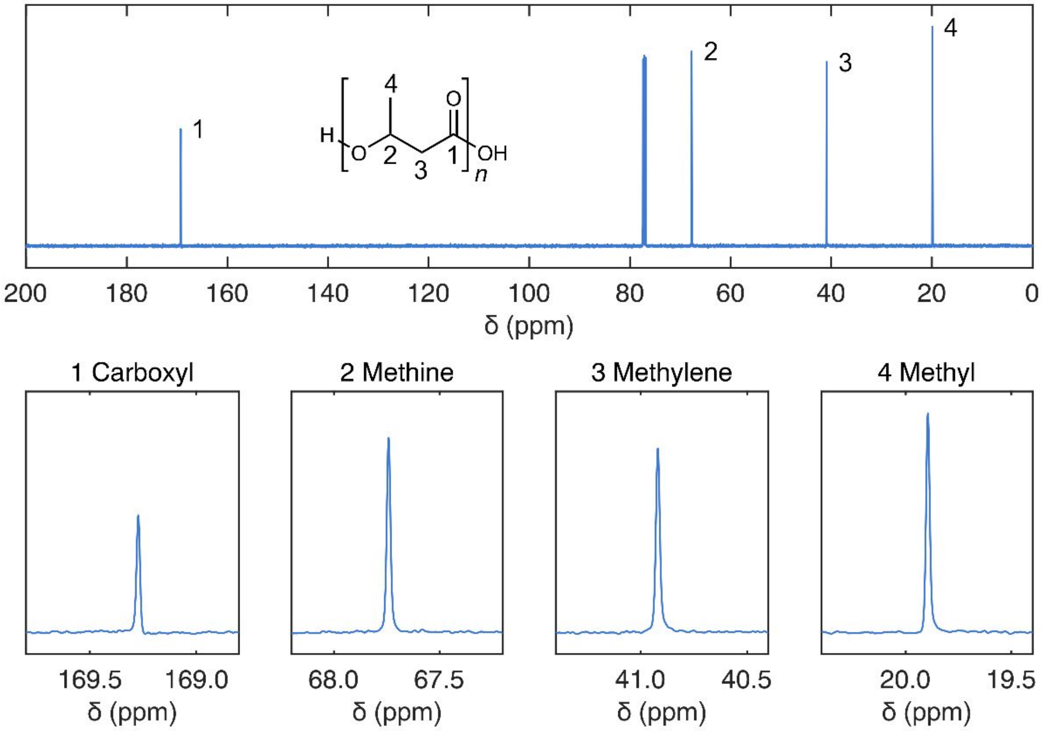

Supplementary Figure 9: ^{13}C NMR spectrum of commercial isotactic PHB. 1 Carboxyl carbon: 169.27 ppm; 2 methine carbon: 67.74 ppm; 3 methylene carbon: 40.92 ppm; 4 methyl carbon: 19.90 ppm

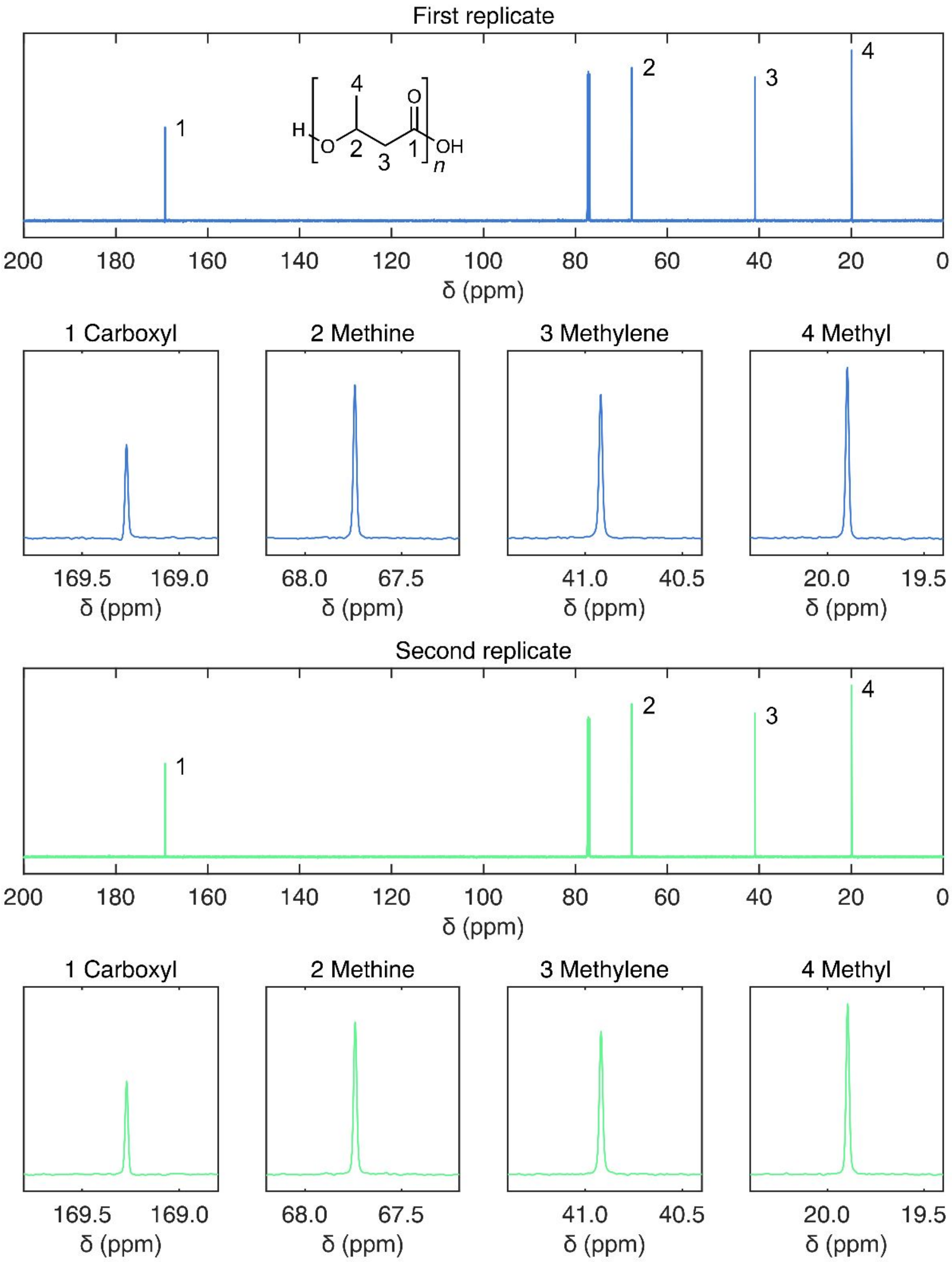

Supplementary Figure 10: ^{13}C NMR spectra of microbial isotactic PHB produced by strain A. 1 Carboxyl carbon: 169.27 ppm; 2 methine carbon: 67.74 ppm; 3 methylene carbon: 40.92 ppm; 4 methyl carbon: 19.90 ppm

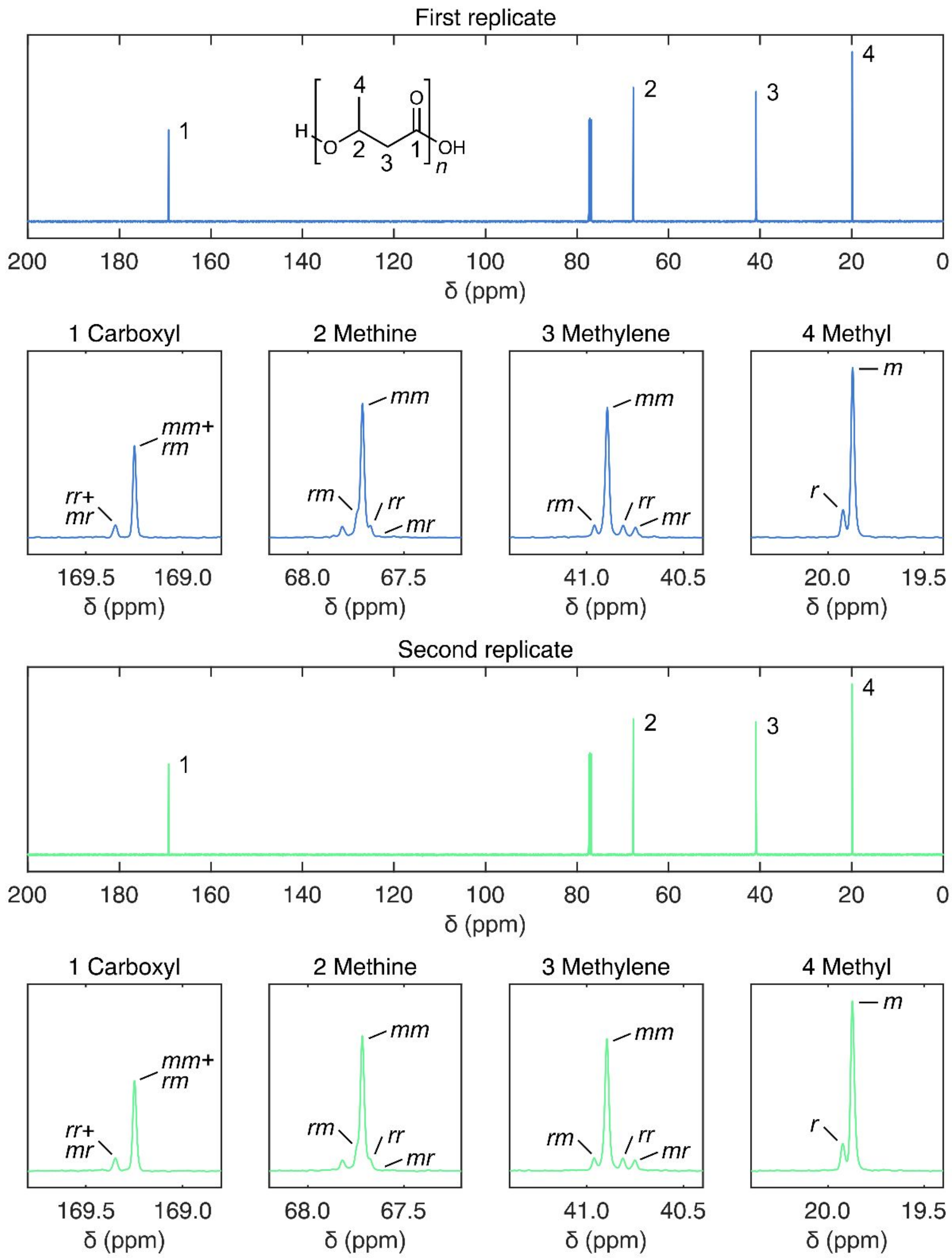

Supplementary Figure 11: ^{13}C NMR spectra of microbial stereoirregular PHB produced by strain B and peak splitting into diads and triads. Diad and triad assignment according to [4]. 1 Carboxyl carbon: 169.36 ppm (*rr* + *mr*), 169.26 ppm (*mm* + *rm*); 2 methine carbon: 67.75 ppm (*rm*), 67.72 ppm (*mm*), 67.78 ppm (*rr*), 67.65 ppm (*mr*); 3 methylene carbon: 40.97 ppm (*rm*), 40.90 ppm

(*mm*), 40.82 ppm (*rr*), 40.76 ppm (*mr*); 4 methyl carbon: 19.93 ppm (*r*), 19.88 ppm (*m*); *m*, meso; *r*, racemo

Supplementary Table 5: Structural, thermal, and mechanical properties of PHB. Data are given as mean and standard deviation. (*S*)-HB, (*S*)-3-hydroxybutyrate fraction; f_r, racemo diad fraction; M_n, number average molar mass; M_w, weight average molar mass; *Đ*, molar mass dispersity; T_g, glass transition temperature; T_m, melting temperature; ΔC_p, change in specific heat capacity of glass transition; ΔH_f, enthalpy of fusion; ΔH_c, enthalpy of crystallization; X_c, degree of crystallinity; *E*, Young's modulus; *σ*, tensile strength; *ε*, elongation at break; *W*, fracture work; *n*, sample size

	Commercial	Strain A	Strain B	*n*
(*S*)-HB (%)	0.00 ± 0.00	0.00 ± 0.00	6.84 ± 0.04	4
f_r (%)	0.0 ± 0.0	0.0 ± 0.0	11.8 ± 0.7	2
M_n (10^4 g mol^{-1})	34 ± 2	36 ± 4	25 ± 2	≥ 6
M_w (10^4 g mol^{-1})	126 ± 15	141 ± 18	66 ± 6	≥ 6
Đ	3.7 ± 0.5	4.0 ± 0.7	2.7 ± 0.2	≥ 6
T_g (°C)	5.7 ± 1.3	8.2 ± 1.5	6.7 ± 0.5	≥ 3
T_m (°C)	174.7 ± 1.3	179.3 ± 0.7	154.3 ± 1.1	≥ 3
ΔC_p (J g^{-1} K^{-1})	0.04 ± 0.06	0.13 ± 0.01	0.70 ± 0.06	≥ 3
ΔH_f (J g^{-1})	−89 ± 4	−85 ± 3	−50 ± 3	≥ 3
ΔH_c (J g^{-1})	80 ± 1	79 ± 3	57 ± 5	≥ 3
X_c (%)	54.7 ± 0.7	54.0 ± 1.7	39.1 ± 3.2	≥ 3
E (GPa)	1.2 ± 0.3	1.1 ± 0.2	0.6 ± 0.1	≥ 9
σ (MPa)	40 ± 1	36 ± 2	36 ± 1	≥ 9
ε (%)	5.3 ± 1.3	4.8 ± 1.0	10.5 ± 1.5	≥ 9
W (MJ m^{-3})	1.4 ± 0.4	1.1 ± 0.3	2.5 ± 0.4	≥ 9

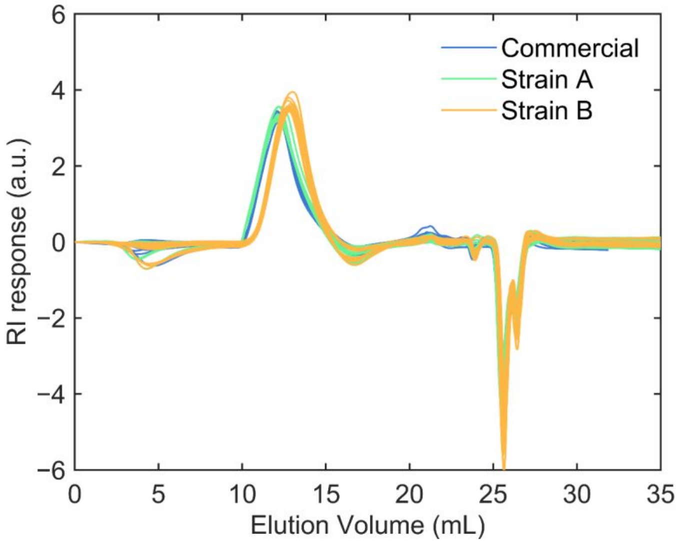

Supplementary Figure 12: Size exclusion chromatography traces of PHB. $n \geq 6$

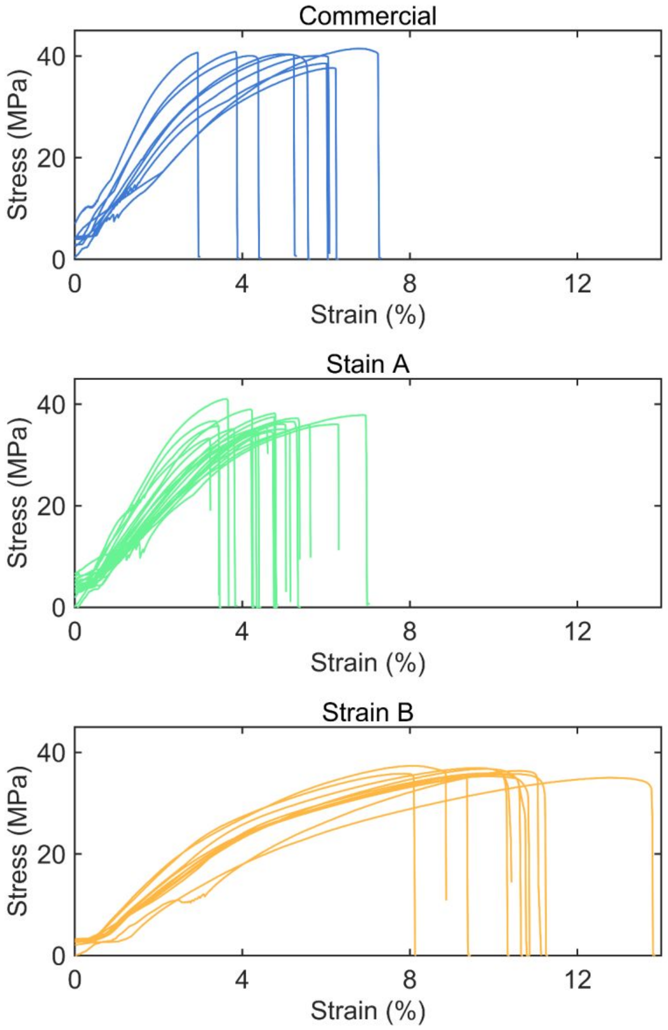

Supplementary Figure 13: Stress–strain curves of tensile test specimens prepared from PHB samples.

Supplementary Description 5: Cause of necessity for higher processing temperature of stereoirregular PHB with prolonged processing cycles.

We believe that at the processing temperature for stereoirregular PHB, melting occurs differently between two polymer phases. Crystalline PHB in amorphous (*S*)-enantiomer rich regions melts at lower temperatures than highly crystalline (*R*)-enantiomer rich regions, which over time leads to separation of the melt peak into two (Supplementary Figure 11). Since the production of discs for both stereoirregular and isotactic PHB was performed slightly below the melting point, cold crystallization appears to have been favored. Naturally, this affected the isotactic PHB, which already forms large parts of its crystalline structure upon cooldown less than the stereoirregular PHB, which showed significant amounts of cold crystallization (Figure 5e). To be able to produce discs, increasing temperatures are required to melt the crystalline regions of the polymer. Nevertheless, it should be noted that the required melting temperature did not reach values similar to isotactic PHB and was still outside of the critical window of molar mass decomposition. This is analogous to the description by [5] for poly[(*R*)-3-hydroxybutyrate-*co*-(*R*)-3-hydroxyvalerate].

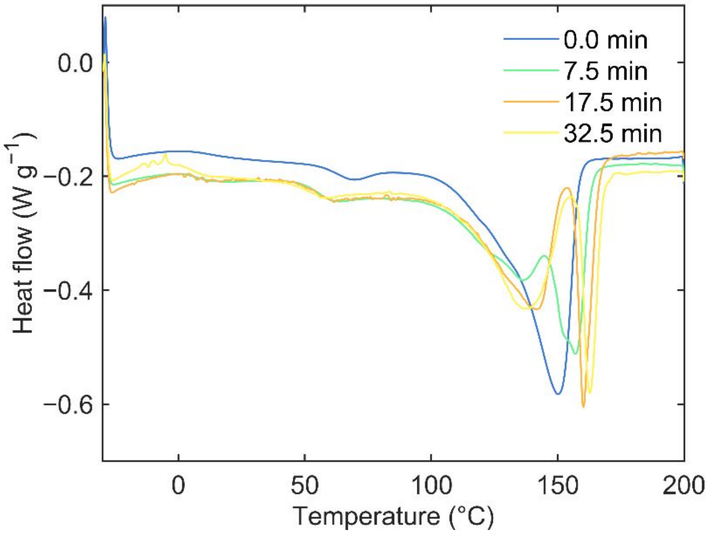

Supplementary Figure 14: Thermal behavior of stereoirregular PHB from strain B after different absolute processing times.

Supplementary Table 6: Structural, thermal and mechanical comparison between microbially and chemically produced stereoirregular PHB. (*S*)-HB, (*S*)-3-hydroxybutyrate fraction; f_r, racemo diad fraction; M_n, number average molar mass; M_w, weight average molar mass; *Đ*, molar mass dispersity; T_g, glass transition temperature; T_m, melting temperature; X_c, degree of crystallinity; *E*, Young's modulus; *σ*, tensile strength; *ε*, elongation at break; *W*, fracture work; n.a., not available

	This work	Bloembergen (1989)[6]	Abe (1994)[7]	Haslböck (2018 & 2019)[8,9,a]
Origin	Microbial	Chemical	Chemical	Chemical
(*S*)-HB (%)	6.84 ± 0.04	n.a.	n.a.	7
f_r (%)	11.8 ± 7	15	16	13
M_n (10^4 g mol^{-1})	34 ± 2	n.a.	11	9.3 ± 1.3
M_w (10^4 g mol^{-1})	126 ± 15	n.a.	20	n.a.
Đ	3.7 ± 0.5	n.a.	1.8	1.12 ± 0.05
T_g (°C)	6.7 ± 0.5	5	6	5 ± 2
T_m (°C)	154.3 ± 1.1	165	132	141 ± 8
X_c (%)	39.1 ± 3.2	34	42	38.6 ± 0.4
E (GPa)	0.6 ± 0.1	n.a.	1.2	1.5 ± 0.1
σ (MPa)	36 ± 1	n.a	15	30 ± 1
ε (%)	10.5 ± 1.5	n.a.	7	5 ± 2
W (MJ m^{-3})	2.5 ± 0.4	n.a.	n.a.	0.47 ± 0.07

[a] Data is best compared against these two publications because the data set is complete and data was acquired on the same instruments.

Supplementary Table 7: *Escherichia coli* strains used in this work.

Strain	Genotype	Source
XL1-Blue	*recA*1 *endA*1 *gyrA*96 *thi*-1 *hsdR*17 *supE*44 *relA*1 *lac* [F′ *proAB* $lacI^q$ZΔM15 *Tn*10 (Tet^R)]	Stratagene
BL21(DE3)	F^- *ompT* $hsdS_B$ (r_B^-, m_B^-) *gal dcm* (DE3)	Thermo Fisher Scientific
BW25113	F^- Δ(*araD-araB*)567 *lacZ*4787Δ::*rrnB*-3 λ^- *rph*-1 Δ(*rhaD-rhaB*)568 *hsdR*514	Datsenko & Wanner (2000)[10]
JW3822	F^- Δ(*araD-araB*)567 *lacZ*4787Δ::*rrnB*-3 λ^- *rph*-1 Δ(*rhaD-rhaB*)568 *hsdR*514 Δ*fadB*1::Kan^R	Baba et al. (2006)[11]
JW2338	F^- Δ(*araD-araB*)567 *lacZ*4787Δ::*rrnB*-3 λ^- *rph*-1 Δ(*rhaD-rhaB*)568 *hsdR*514 Δ*fadJ*1::Kan^R	Baba et al. (2006)[11]
BW25113 Δ*fadB*	F^- Δ(*araD-araB*)567 *lacZ*4787Δ::*rrnB*-3 λ^- *rph*-1 Δ(*rhaD-rhaB*)568 *hsdR*514 Δ*fadB*1	This work
BW25113 Δ*fadB* Δ*fadJ*::Kan^R	F^- Δ(*araD-araB*)567 *lacZ*4787Δ::*rrnB*-3 λ^- *rph*-1 Δ(*rhaD-rhaB*)568 *hsdR*514 Δ*fadB*1 Δ*fadJ*1::Kan^R	This work
BW25113 Δ*fadB* Δ*fadJ*	F^- Δ(*araD-araB*)567 *lacZ*4787Δ::*rrnB*-3 λ^- *rph*-1 Δ(*rhaD-rhaB*)568 *hsdR*514 Δ*fadB*1 Δ*fadJ*1	This work

Supplementary Table 8: Plasmids used in this work.

Plasmid	Description	Source
CBR_P_1189	Plasmid encoding *phaC1* from *Pseudomonas* sp. 61-3	Janine Simon (internal)
CBR_P_1232	Plasmid for heterologous protein production of *hbd* from *Clostridium acetobutylicum*	Janine Simon (internal)
pACYC184	Low copy number cloning plasmid	ATCC #37033
pACYC-CkHbd	Tetracycline resistance gene of pACYC184 replaced by *hbd* from *Clostridium kluyveri* DSM 555	This work
pACYC-sfGFP	Tetracycline resistance gene of pACYC184 replaced by super-folder *gfp* from *Aequorea victoria*	This work
pCkHbd	Plasmid for heterologous protein production of *hbd* from *Clostridium kluyveri* DSM 555	This work
pCP20	Temperature inducible plasmid for FLP recombination	Cherepanov et al. (1995)[12]
pET-28a(+)	Expression vector from the pET system for restriction–ligation cloning	Novagen
pET28a_BsaI	pET-28a(+) derivative for Golden Gate cloning	Rohweder et al. (2018)[13]
pUC19	High copy number cloning plasmid	Thermo Fisher Scientific
pUC19-Kan	Ampicillin resistance replaced by kanamycin resistance of pET-28a(+)	This work
pUC-Kan-AvPhaEC-CkHbd-CnPhaAB	*hbd* from *Clostridium kluyveri* DSM 555 placed before *phaA* in pUC-Kan-AvPhaEC-CnPhaAB	This work
pUC-Kan-AvPhaEC-CnPhaAB	*phaC* of pUC-Kan-CnPhaCAB replaced by *phaEC* from *Allochromatium vinosum* DSM 180	This work
pUC-Kan-AvPhaEC-CnPhaA-CkHbd-CnPhaB	*hbd* from *Clostridium kluyveri* DSM 555 placed before *phaB* in pUC-Kan-AvPhaEC-CnPhaAB	This work
pUC-Kan-CkHbd-AvPhaEC-CnPhaAB	*hbd* from *Clostridium kluyveri* DSM 555 placed before *phaEC* in pUC-Kan-AvPhaEC-CnPhaAB	This work
pUC-Kan-CkHbd-CsPhaC-CnPhaAB	*hbd* from *Clostridium kluyveri* DSM 555 placed before *phaC* in pUC-Kan-CsPhaC-CnPhaAB	This work
pUC-Kan-CnPhaCAB	Plasmid encoding *phaCAB* operon from *Cupriavidus necator* H16 controlled by native promoter	This work

pUC-Kan-CsPhaC-CkHbd-CnPhaAB	*hbd* from *Clostridium kluyveri* DSM 555 placed before *phaA* in pUC-Kan-CsPhaC-CnPhaAB	This work
pUC-Kan-CsPhaC-CnPhaAB	*phaC* of pUC-Kan-CnPhaCAB replaced by *phaC* from *Chromobacterium* sp. USM2	This work
pUC-Kan-CsPhaC-CnPhaA-CkHbd-CnPhaB	*hbd* from *Clostridium kluyveri* DSM 555 placed before *phaB* in pUC-Kan-CsPhaC-CnPhaAB	This work
pUC-Kan-PmPhaRC-CnPhaAB	*phaC* of pUC-Kan-CnPhaCAB replaced by *phaRC* from *Priestia megaterium* 22-2	This work
pUC-Kan-PsPhaC1-CnPhaAB	*phaC* of pUC-Kan-CnPhaCAB replaced by *phaC* from *Pseudomonas* sp. 61-3	This work
pYTK047	Plasmid encoding super-folder green fluorescent protein	Lee et al. (2015)[14]

References

(1) Karp, P. D.; Paley, S.; Caspi, R.; Kothari, A.; Krummenacker, M.; Midford, P. E.; Moore, L. R.; Subhraveti, P.; Gama-Castro, S.; Tierrafria, V. H.; Lara, P.; Muñiz-Rascado, L.; Bonavides-Martinez, C.; Santos-Zavaleta, A.; Mackie, A.; Sun, G.; Ahn-Horst, T. A.; Choi, H.; Covert, M. W.; Collado-Vides, J.; Paulsen, I. The EcoCyc Database (2023). *EcoSal Plus* **2023**, *11* (1), eesp00022023. DOI: 10.1128/ecosalplus.esp-0002-2023. Published Online: May. 11, 2023.

(2) Ishikawa, M.; Tsuchiya, D.; Oyama, T.; Tsunaka, Y.; Morikawa, K. Structural basis for channelling mechanism of a fatty acid beta-oxidation multienzyme complex. *EMBO J.* **2004**, *23* (14), 2745–2754. DOI: 10.1038/sj.emboj.7600298. Published Online: Jul. 1, 2004.

(3) van Wegen, R. J.; Lee, S.-Y.; Middelberg, A. P. J. Metabolic and kinetic analysis of poly(3-hydroxybutyrate) production by recombinant *Escherichia coli*. *Biotechnol. Bioeng.* **2001**, *74* (1), 70–81. DOI: 10.1002/bit.1096.

(4) Huang, H.-Y.; Xiong, W.; Huang, Y.-T.; Li, K.; Cai, Z.; Zhu, J.-B. Spiro-salen catalysts enable the chemical synthesis of stereoregular polyhydroxyalkanoates. *Nat. Catal.* **2023**, *6* (8), 720–728. DOI: 10.1038/s41929-023-01001-7.

(5) Bossu, J.; Le Moigne, N.; Dieudonné-George, P.; Dumazert, L.; Guillard, V.; Angellier-Coussy, H. Impact of the processing temperature on the crystallization behavior and mechanical properties of poly[*R*-3-hydroxybutyrate-*co*-(*R*-3-hydroxyvalerate)]. *Polymer* **2021**, *229*, 123987. DOI: 10.1016/j.polymer.2021.123987.

(6) Bloembergen, S.; Holden, D. A.; Bluhm, T. L.; Hamer, G. K.; Marchessault, R. H. Stereoregularity in synthetic β-hydroxybutyrate and β-hydroxyvalerate homopolyesters. *Macromolecules* **1989**, *22* (4), 1656–1663. DOI: 10.1021/ma00194a027.

(7) Abe, H.; Matsubara, I.; Doi, Y.; Hori, Y.; Yamaguchi, A. Physical Properties and Enzymic Degradability of Poly(3-hydroxybutyrate) Stereoisomers with Different Stereoregularities. *Macromolecules* **1994**, *27* (21), 6018–6025. DOI: 10.1021/ma00099a013.

(8) Haslböck, M.; Klotz, M.; Steiner, L.; Sperl, J.; Sieber, V.; Zollfrank, C.; van Opdenbosch, D. Structures of Mixed-Tacticity Polyhydroxybutyrates. *Macromolecules* **2018**, *51* (14), 5001–5010. DOI: 10.1021/acs.macromol.8b01047.

(9) Haslböck, M.; Klotz, M.; Sperl, J.; Sieber, V.; Zollfrank, C.; van Opdenbosch, D. Mechanical and Thermal Properties of Mixed-Tacticity Polyhydroxybutyrates and Their Association with Iso- and Atactic Chain Segment Length Distributions. *Macromolecules* **2019**, *52* (14), 5407–5418. DOI: 10.1021/acs.macromol.9b00931.

(10) Datsenko, K. A.; Wanner, B. L. One-step inactivation of chromosomal genes in *Escherichia coli* K-12 using PCR products. *Proc. Natl. Acad. Sci. U. S. A.* **2000**, *97* (12), 6640–6645. DOI: 10.1073/pnas.120163297.
(11) Baba, T.; Ara, T.; Hasegawa, M.; Takai, Y.; Okumura, Y.; Baba, M.; Datsenko, K. A.; Tomita, M.; Wanner, B. L.; Mori, H. Construction of *Escherichia coli* K-12 in-frame, single-gene knockout mutants: the Keio collection. *Mol. Syst. Biol.* **2006**, *2*, 2006.0008. DOI: 10.1038/msb4100050. Published Online: Feb. 21, 2006.
(12) Cherepanov, P. P.; Wackernagel, W. Gene disruption in *Escherichia coli*: Tc^R and Km^R cassettes with the option of Flp-catalyzed excision of the antibiotic-resistance determinant. *Gene* **1995**, *158* (1), 9–14. DOI: 10.1016/0378-1119(95)00193-a.
(13) Rohweder, B.; Semmelmann, F.; Endres, C.; Sterner, R. Standardized cloning vectors for protein production and generation of large gene libraries in *Escherichia coli*. *BioTechniques* **2018**, *64* (1), 24–26. DOI: 10.2144/000114628. Published Online: Jan. 1, 2018.
(14) Lee, M. E.; DeLoache, W. C.; Cervantes, B.; Dueber, J. E. A Highly Characterized Yeast Toolkit for Modular, Multipart Assembly. *ACS Synth. Biol.* **2015**, *4* (9), 975–986. DOI: 10.1021/sb500366v. Published Online: May. 1, 2015.
